# Strategic deployment of feature-based attentional gain in primate visual cortex

**DOI:** 10.1371/journal.pbio.3000387

**Published:** 2019-08-06

**Authors:** Vladislav Kozyrev, Mohammad Reza Daliri, Philipp Schwedhelm, Stefan Treue

**Affiliations:** 1 Cognitive Neuroscience Laboratory, German Primate Center–Leibniz Institute for Primate Research, Goettingen, Germany; 2 Bernstein Center for Computational Neuroscience, Goettingen, Germany; 3 Laboratory of Systems Neuroscience and Imaging in Psychiatry (SNIP), University Medical Center Goettingen, Germany; 4 Department of Cognitive Neurology, University Medical Center Goettingen, Germany; 5 Neuroscience and Neuroengineering Research Lab., Biomedical Engineering Department, School of Electrical Engineering, Iran University of Science and Technology (IUST), Narmak, Tehran, Iran; 6 Cognitive Neurobiology Lab., School of Cognitive Sciences (SCS), Institute for Research in Fundamental Sciences (IPM), Niavaran, Tehran, Iran; 7 Center for Mind and Brain Sciences, University of Trento, Italy; 8 Institute of Molecular and Clinical Ophthalmology Basel (IOB), Switzerland; 9 Functional Imaging Laboratory, German Primate Center–Leibniz Institute for Primate Research, Goettingen, Germany; 10 Leibniz ScienceCampus PrimateCognition, Goettingen, Germany; 11 Faculty of Biology and Psychology, University of Goettingen, Germany; Yeshiva University Albert Einstein College of Medicine, UNITED STATES

## Abstract

Attending to visual stimuli enhances the gain of those neurons in primate visual cortex that preferentially respond to the matching locations and features (on-target gain). Although this is well suited to enhance the neuronal representation of attended stimuli, it is nonoptimal under difficult discrimination conditions, as in the presence of similar distractors. In such cases, directing attention to neighboring neuronal populations (off-target gain) has been shown to be the most efficient strategy, but although such a strategic deployment of attention has been shown behaviorally, its underlying neural mechanisms are unknown. Here, we investigated how attention affects the population responses of neurons in the middle temporal (MT) visual area of rhesus monkeys to bidirectional movement inside the neurons’ receptive field (RF). The monkeys were trained to focus their attention onto the fixation spot or to detect a direction or speed change in one of the motion directions (the “target”), ignoring the distractor motion. Population activity profiles were determined by systematically varying the patterns’ directions while maintaining a constant angle between them. As expected, the response profiles show a peak for each of the 2 motion directions. Switching spatial attention from the fixation spot into the RF enhanced the peak representing the attended stimulus and suppressed the distractor representation. Importantly, the population data show a direction-dependent attentional modulation that does not peak at the target feature but rather along the slopes of the activity profile representing the target direction. Our results show that attentional gains are strategically deployed to optimize the discriminability of target stimuli, in line with an optimal gain mechanism proposed by Navalpakkam and Itti.

## Abstract 2

This study documents the neural basis of a sophisticated attentional system that is able to differentially distribute feature-based attention such that the strongest attentional modulation is directed towards those neurons that are the most informative contributors to the task at hand.

## Introduction

In a natural environment, the visual system is typically challenged with cluttered input, containing a variety of stimuli that need to be segregated for further processing. This task is complicated by input that has overlapping properties in both retinotopic location and stimulus features, such as colors, shapes, or motion directions. Yet, a robust and fast discrimination of single stimuli in this torrent of information is paramount, because specific (and often small) parts of the input may bear great behavioral relevance for the organism.

Consider a monkey moving through a tree canopy, watching a predator behind the leaves. Here, it is important to segregate behaviorally irrelevant, possibly obscuring visual input (the leaves) from the behaviorally relevant stimulus behind them. On the level of individual neurons, attention supports this selective enhancement by increasing the gain of specific cells tuned to the properties of the relevant stimulus [1–6]. Thus, attention is thought to propagate behaviorally relevant information by enhancing the activity of those cells that are maximally selective for the stimulus at hand (on-target gain). However, when targets and distractors are similar, on-target enhancement is not always optimal for the behavioral strategy. Theoretical, psychophysical, and imaging studies [7–10] suggest that in those cases neuronal populations tuned away from the attended stimulus are the best targets for enhancement because they can better discriminate neighboring features (off-target gain). Such a behaviorally advantageous modulation of activity gains has not yet been shown at the single neuron level.

In the present study, we determined how attention selectively modulates representations of 2 close-by stimuli, both presented in the receptive field (RF) of individual units recorded from motion-selective, extrastriate middle temporal (MT) area of 2 macaque monkeys. The 2 stimuli differed in their featural properties only through a constant direction difference of 120°. The animals were cued to solve a task on only 1 of the 2 directions and ignore the other. A selective modulation of each stimulus representation (based on its behavioral relevance) could then be accomplished with the help of feature-based attention (FBA). Previous studies using 2 moving stimuli in MT RFs [11–15] focused on how attention removes the influence of a distractor on the neural response to the target, typically using only 2 opposite directions for the 2 stimuli. We instead swept through a full set of directions while keeping the featural distance between the 2 stimuli constant to recover not only the response of neurons preferring 1 of the 2 stimuli but the full population response profile of MT to such a target-distractor pair.

Such a visual input has been shown to produce bilobed response profiles across MT neurons with each response peak corresponding to one of the 2 (i.e., the behaviorally relevant and irrelevant) stimuli [16,17]. We then investigated the effects of attention on the response profile and found that the behavioral relevance of each stimulus shaped its individual response peak. Further, we estimated to which directions attention was likely deployed, relative to the displayed set of stimuli. Using computational modeling based on the feature-similarity gain model [18], we show that FBA can be allocated in a highly task-specific manner, optimized to best enhance the discriminability of the target stimulus, in line with an optimal off-target gain mechanism [7].

## Materials and methods

Research with nonhuman primates represents a small but indispensable component of neuroscience research. The scientists in this study are aware and are committed to the great responsibility they have in ensuring the best possible science with the least possible harm to the animals [19].

The core approach of our study was to record full tuning curves of neuronal responses to the combination of 2 directions (forming a constant angle) within the RF in order to estimate the population response profile to such stimuli under different attentional conditions. We used bidirectional motion patterns made up of 2 adjacent but spatially separated patterns of random dots. Their individual motion directions always formed a relative angle of 120°. Both apertures were placed in the classical RF of the current MT neuron. To estimate the full tuning curve to such bidirectional stimulation, the joint motion direction of the patterns was varied in 30° steps.

### Monkey training and surgery

Two male rhesus monkeys (*Macaca mulatta*) were trained to perform visual attentional tasks. The animals were implanted with a custom-made titanium post to prevent head movements during training and recording, and a recording chamber (Crist Instruments, Hagerstown, MD) on top of a craniotomy over the left (monkey C) or the right (monkey H) parietal lobe. The chamber positions were based on anatomical MRI scans. Surgeries were performed aseptically under isoflurane anesthesia using standard techniques (see [18]) including appropriate peri-surgical analgesia and monitoring.

### Ethics statement

All animal procedures of this study have been approved by the responsible regional government office (Niedersaechsisches Landesamt fuer Verbraucherschutz und Lebensmittelsicherheit [LAVES]) under the permit numbers 33.42502/08-07.02 and 33.14.42502-04-064/07. We have established a comprehensive set of measures to ensure that the severity of our experimental procedures falls into the category of mild to moderate, according to the severity categorization of Annex VIII of the European Union’s directive 2010/63/EU on the protection of animals used for scientific purposes (see also [20]).

The animals were group-housed with other macaque monkeys in facilities of the German Primate Center in Goettingen, Germany, in accordance with all applicable German and European regulations. The facility provides the animals with an enriched environment (including a multitude of toys, wooden structures, and other enrichment; [21,22]), natural as well as artificial light, and a space exceeding the size requirements of the European regulations, including access to outdoor space. The German Primate Center has several staff veterinarians that monitor and examine the animals and consult on procedures. During the study, the animals had unrestricted access to food and fluid, except for the days when data were collected or the animal was trained on the behavioral paradigm. On these days, the animals were allowed unlimited access to fluid through their performance in the behavioral paradigm. Here, the animals received fluid rewards for every correctly performed trial. Throughout the study, the animals' psychological and medical welfare was monitored by the veterinarians, the animal facility staff, and the lab’s scientists, all specialized in working with nonhuman primates. The 2 animals were healthy at the conclusion of our study and were used in follow-up studies.

### Experimental procedure

Single-unit action potentials were recorded extracellularly with single tungsten electrodes (FHC, Bowdoinham, ME) after penetration of the dura with a sharp guide tube. The electrode was advanced using a hydraulic micropositioner (David Kopf Instruments, Tujunga, CA). Electrode impedances ranged from 0.5 to 2.8 MΩ. Neuronal activity was amplified and filtered (bandpass 150–5,000 Hz). Action potentials of the majority of recorded units were sorted online using a Plexon MAP data acquisition system (Plexon, Dallas, TX). In the first recording sessions, action potentials were isolated using a window discriminator (BAK Electronics, Mount Airy, MD). Area MT was identified by its anatomical position, the high proportion of direction-selective cells, and the typical size-eccentricity relationship of RFs. Eye positions were monitored using a video-based eye tracking system (ET-49; Thomas Recording, Giessen, Germany). Eye positions were sampled at 230 Hz, digitized, and stored at 200 Hz. Gaze direction was controlled during the recordings to stay within a window of 1.2° radius around the fixation spot; trials in which the gaze direction left that window were excluded from the analysis.

### Visual stimuli

The experiments were conducted using an Apple Macintosh computer running custom software and a Sony Trinitron (22 in) monitor with 75 Hz refresh rate. The monkey viewed the display binocularly in a dimly lit room from a distance of 57 cm. The spatial resolution of the display was 40 pixels per degree of visual angle. The shape of the RF, as well as its preferred direction and speed, were estimated in a separate mapping and tuning session performed before the main task (see S1 Text and S1 Fig).

**Fig 1:**
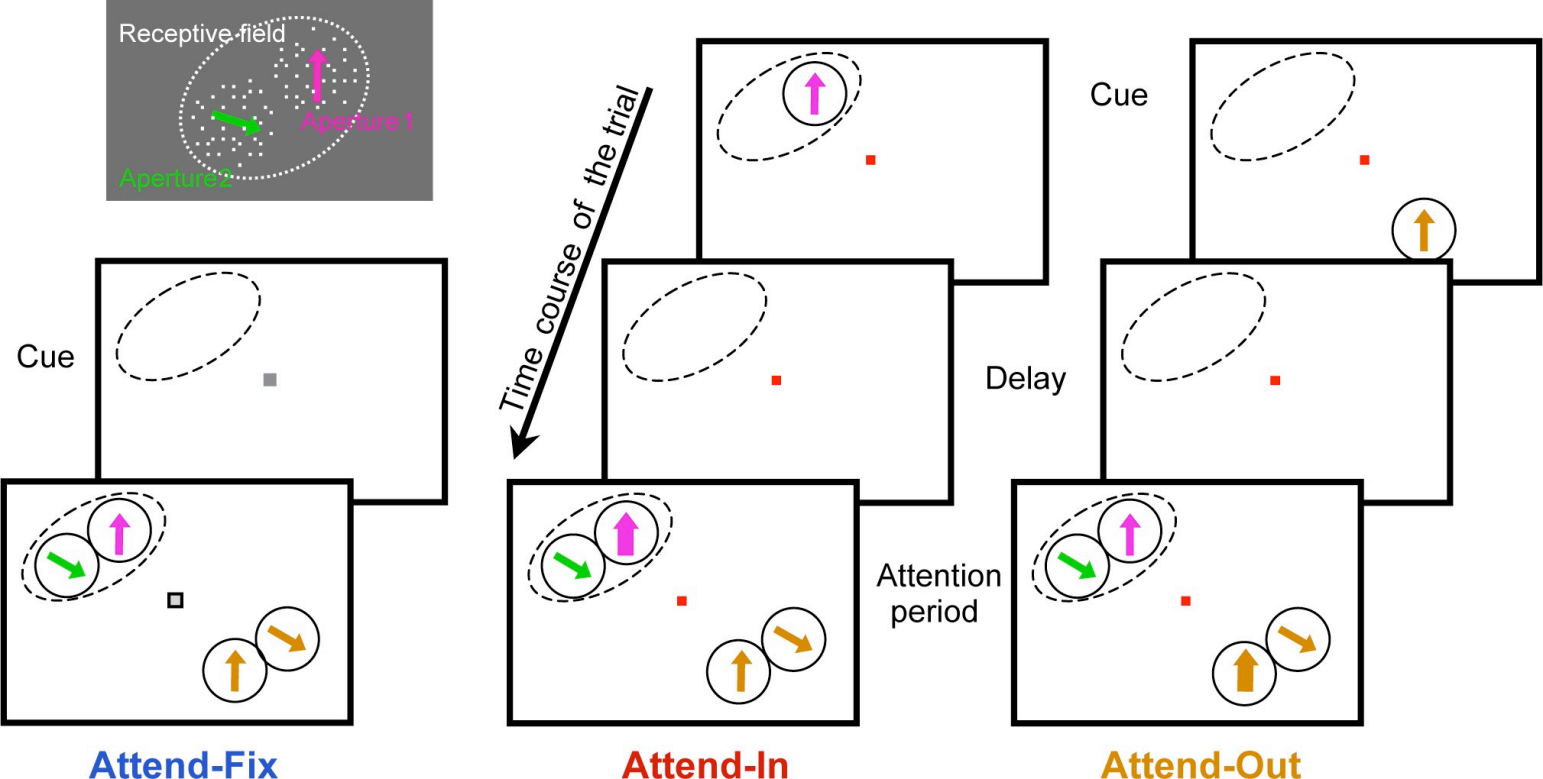
Visual stimulus and behavioral task. The panel in the upper left shows an example of the stimulus layout in the RF with the luminance polarity used in the experiment. We used bidirectional motion patterns composed of 2 adjacent but spatially separated RDP that moved within 2 stationary virtual apertures placed within the classical RF of a mapped MT neuron (white dashed line). The dots were white, on a gray background. Directions of the movement were systematically varied, keeping the angle between them constant and equal to 120°. In the attend-fix condition (left panel), the monkey was trained to detect a luminance change in the fixation spot while 2 motion components were presented in the RF and 2 other components outside the RF. The directions of the latter were randomly picked. In the other 2 conditions, the same visual stimuli were shown inside the RF but the monkey was cued either to one of the patterns (the right one, as depicted here) within the RF (attend-in condition, middle panel) or to a pattern outside the RF (attend-out condition, right panel), while maintaining its gaze on the fixation spot throughout each trial. The cue (marked by arrows: magenta in the middle top panel and orange in the right one) appeared at the same location and moved in the same direction as the target. Both the target (marked by a thicker arrow) and distractor patterns appeared simultaneously after a delay. The monkey was required to detect a transient change of either speed or direction of motion in the target RDP. MT, middle temporal; RDP, random dot pattern; RF, receptive field.

The bidirectional stimuli used in the main task were 2 random dot patterns (RDPs) presented within stationary adjacent virtual apertures matching the excitatory part of the RF (a sketch is shown in the upper left insertion of Fig 1). Another pair of RDPs was presented far outside the RF (in the opposite visual hemifield symmetrically to the first pair in respect to the fixation point). Each RDP had a density of 10 dots per square degree. The width of each dot was 6 min of arc. All dots were white (luminance 85 cd/m^2^) and were displayed on a gray background (luminance 15 cd/m^2^). The basic speed of the dots in the RDP was matched to the preferred speed of the neuron and usually was between 4 and 16°/sec. The 12 directions of the patterns used to recover the tuning curve were chosen such that one of them was well aligned with the neuron’s preferred direction. Simultaneous isolation of 2 units was sometimes possible, and both units were recorded if the 2 RFs showed enough overlap to allow for the proper placement of the stimuli for both neurons. In this case, the stimuli were matched to the preferred speed of the more robust or better isolated neuron.

### Behavioral task

A fixation spot (a red square of 12 min of arc width) appeared in the middle of the screen before each trial. The monkeys were trained to maintain their gaze on the fixation spot throughout each trial. The monkey initiated a trial by touching a bar. Immediately afterwards a cue appeared to specify a target stimulus (see below). The target, which could be either a RDP or the fixation spot, appeared later during the trial together with several distracting RDPs. The monkey had to detect a transient change in the target and respond by releasing the lever within 150 to 650 ms after the change. Depending upon the type, location, and feature of the attention target, as well as the number of patterns in the RF, the following experimental conditions were used (see Fig 1).

In the attend-fix condition, the color of the fixation spot changed from red to gray immediately after the monkey pressed the lever. After 600 ms (400 ms in the first few sessions), several distractor patterns appeared inside and outside the RF, and each of them could contain a change at a random time between 800 and 2,400 ms after their onset. The monkeys were required to detect a luminance change (from 85 to 52 cd/m^2^ for 130 ms) in the fixation spot, which took place within the same time window. For all cells, we collected data when 2 patterns were simultaneously present in the RF. For the majority of recorded neurons in additional trials, we presented only 1 pattern in the RF (at 1 of 2 locations used for the bidirectional stimulation). This unidirectional stimulation served to check if the 2 apertures were properly positioned within the RF to elicit similar response strengths.

In the attend-in and attend-out conditions, the fixation spot remained red during the whole trial. The cue (a moving RDP) was presented for 600 ms at the target location and moved in the same direction as the target. After an 800-ms delay, 4 RDPs appeared simultaneously inside and outside the RF: 1 target and 3 distractors. This long delay was chosen to ensure that any adaptation caused by the cue has dissipated [23–25]. Even if still present, the adaptation would have affected primarily the early response epoch of the transient onset response to the appearance of the target stimulus (up to 150 ms; see [25]), which we did not consider by our analysis. Furthermore, it was shown by Kar and Krekelberg [26] that even strong adaptors provide no change or rather sharpening in tuning width of monkey MT neurons, which is opposite to our observations.

The first change of motion within the trial took place between 400 and 1,200 ms after the patterns’ onset and could occur in either the target (“early target change”) or one of the distractors. In the latter case, the target change was presented between 500 and 1,300 ms after the distractor change (“late target change”). In case of the early target change, a change in the distractor was not displayed because the response time window following the target change finished the trial. To ensure that animals allocated attention from the beginning of a trial, trials with an early target change were twice as frequent as trials with late target change (the latter frequency was the same as in the attend-fix trials). The changed motion (direction change by 45° clockwise or counterclockwise or speed increase 67% relative to the original speed) lasted 130 ms, after which the direction or speed returned to their original values. The target change took place either inside the RF (attend-in condition) or far outside the RF (attend-out condition). We did not observe any difference between direction-change (approximately 2/3 of the data) and the speed-change (approximately 1/3 of the data) data in regard to the results presented here. We have therefore pooled the 2 conditions throughout.

Direction of motion in aperture 2 was always 120° clockwise relative to that in aperture 1. In Fig 1 and throughout the paper, aperture 1 is depicted right from aperture 2 (although the real patterns could have any relative position on the screen). For simplicity of our description, we will call patterns in the apertures 1 and 2, respectively, “right” and “left.” To determine a direction-tuning curve for a neuron in a given sensory and behavioral condition, we systematically varied the directions of both motion components in 30° steps. In all cells, full-tuning curves (12 points in each) were determined for 2 bidirectional behavioral conditions (the attend-fix and the attend-in conditions). Recording of the unidirectional case (with attention on the fixation point) provided 2 additional tuning curves. In the attend-fix and the attend-in conditions, the direction of the motion patterns outside the RF was randomized. In the attend-out condition, the target moved either in the preferred or null direction, whereas the stimulus in the RF always moved in the preferred direction. These 2 conditions were included in order to determine the magnitude of FBA modulation [6]. Trials of different attentional conditions and different direction combinations were presented in random order. A data set was excluded from further analysis if the monkey’s performance was below 75% of all trials with unbroken fixation. A complete list of experimental conditions is presented in Table A in S1 Text.

We recorded 67 cells from monkey H and 46 cells from monkey C. All cells of C and 42 cells of H were recorded with the complete set of conditions; the remaining 25 data sets of H did not include the unidirectional attend-fix conditions.

### Data analysis

Our custom software allowed an online preliminary analysis of the recorded spike trains. All further calculations were performed with custom scripts written in MATLAB (The MathWorks, Natick, MA). For each trial we computed a spike density function by convolving the spike train with a Gaussian kernel (σ = 21.2 ms; see S2 Fig for an example). The firing rate for each condition was calculated by averaging the spike density functions of respective trials between 200 and 700 ms after the onset of the RDPs. The MT neurons show robust attentional modulation within that time window, as shown elsewhere [12,27]. A minimum of 2 successful repetitions of each condition was required for the inclusion of a cell into further analysis. In the majority of the included neurons though, each condition was repeated 4 to 7 times (see S3 Fig).

Attentional effects were quantified by computing an attentional index (AI), defined as the difference in firing rates between 2 conditions, divided by their sum [4]:

$$AI=\frac{R_{2}{-R}_{1}}{R_{2}{+R}_{1}},$$ (1)

where *R*_*1*_ and *R*_*2*_ are the firing rates in 2 attentional conditions. Negative values of the index indicate an enhanced response in *R*_*1*_ relative to *R*_*2*_, positive values indicate a higher response in *R*_*2*_. Such an index creates normally distributed values (confirmed for our data using Lillieford’s test), allowing the use of parametric statistics. Standard errors of mean AIs across the set of recorded neurons were calculated in each stimulus condition. In order to express modulation in more intuitive units for the general data description, the average AIs were converted to average attentional ratios (ARs), expressed in percent of response modulation:

$$AR=\frac{2AI}{1-AI}\times 100\%=\frac{R_{2}{-R}_{1}}{R_{1}}\times 100\%.$$ (2)

In order to quantitatively estimate changes in the population activity profile between different conditions, the individual tuning curves were aligned according to their preferred direction and fitted by periodic Gaussian functions [28]. The weighting factors for the fitting were inversely related to the standard errors of the individual data points. See more details on the data analysis in S1 Text.

Responses to the unidirectional conditions (*G*) were fitted by:

$$G=ae^{-\frac{{\left\|\left(x-c\right)\right\|}_{360}^{2}}{2b^{2}}}+d,$$ (3)

where: ‖(*x−c*)‖_*T*_ = *mod*(*x−c*+*T*/2,*T*)−*T*/2, period *T =* 360° for the direction-tuned MT neurons; parameters *a* (amplitude), *b* (standard deviation), *c* (location of maximum), and *d* (asymptote) capture, respectively, the 4 characteristics of a direction-selective cell: the directional gain, the selectivity, the preferred direction, and the response to the antipreferred direction [6]. The bidirectional data recorded in the attend-fix and the attend-in conditions were fitted by a sum of 2 periodic Gaussians (SG) with 7 free parameters corresponding to the independent responses to the 2 stimulus components and a shared asymptote:

$$SG=a_{1}e^{-\frac{{\left\|\left(x-c_{1}\right)\right\|}_{360}^{2}}{2b_{1}^{2}}}+a_{2}e^{-\frac{{\left\|\left(x-c_{2}\right)\right\|}_{360}^{2}}{2b_{2}^{2}}}+d,$$ (4)

where indexes 1 and 2 denote the aperture number of the RF stimulus (right and left patterns, respectively); angular variable *x* is a mean direction of motion between the 2 stimulus components. In our convention, *x* = 0° denotes upward direction and *x* = 90° denotes rightward direction. To exclude cells with poor directional selectivity from the population analysis, we used the 95% confidence intervals to ensure that these for the Gaussian amplitudes did not include 0. So we restricted our approach to cells in which both fitted Gaussians in the attend-fix condition had significant amplitudes. This criterion was not applied in the attend-in condition because the absence of a second Gauss function in the fit might reflect an attentional suppression. In such cases, refitting of the attend-in tuning curve was performed, keeping the location and standard deviation of the nonsignificant Gaussian fixed at the respective values inherited from the attend-fix condition fit. A fit for an example neuron is depicted in S4 Fig.

### Model assessment

We used several approaches to quantify the goodness of our fits. First, we determined the coefficient of determination (*R*^*2*^), which indicates the proportionate amount of variation in the response variable explained by the model. It was adjusted based on the residual degrees of freedom (difference between the number of response values *n* and the number of fitted coefficients *k*) and calculated using the MATLAB Curve Fitting Toolbox:

$$R_{adj}^{2}=1-\left(\frac{n-1}{n-k}\right)\frac{SSE}{SST},$$ (5)

where *SST* is the sum of squared data deviations from the mean, and *SSE* is the sum of squared prediction errors.

Secondly, as another way to compare models with different numbers of free parameters, we employed the Akaike information criterion (AIC; Eq 6; see [29]) and, thirdly, the closely related and even more conservative Bayesian information criterion (BIC; Eq 7):

$$AIC=nln\left(\frac{SSE}{n}\right)+2k;$$ (6)

$$BIC=nln\left(\frac{SSE}{n}\right)+kln(n).$$ (7)

In both equations, *n* = 12 (number of the measured points) and *k ≥*1 (number of free model parameters). These criteria provide normally distributed fit quality estimates for each of the used models (checked by Lillieford test), allowing parametric statistics.

The numerical values used for the figures, including the individual data points, are provided in S1 Data and S2 Data.

## Results

**Fig 2:**
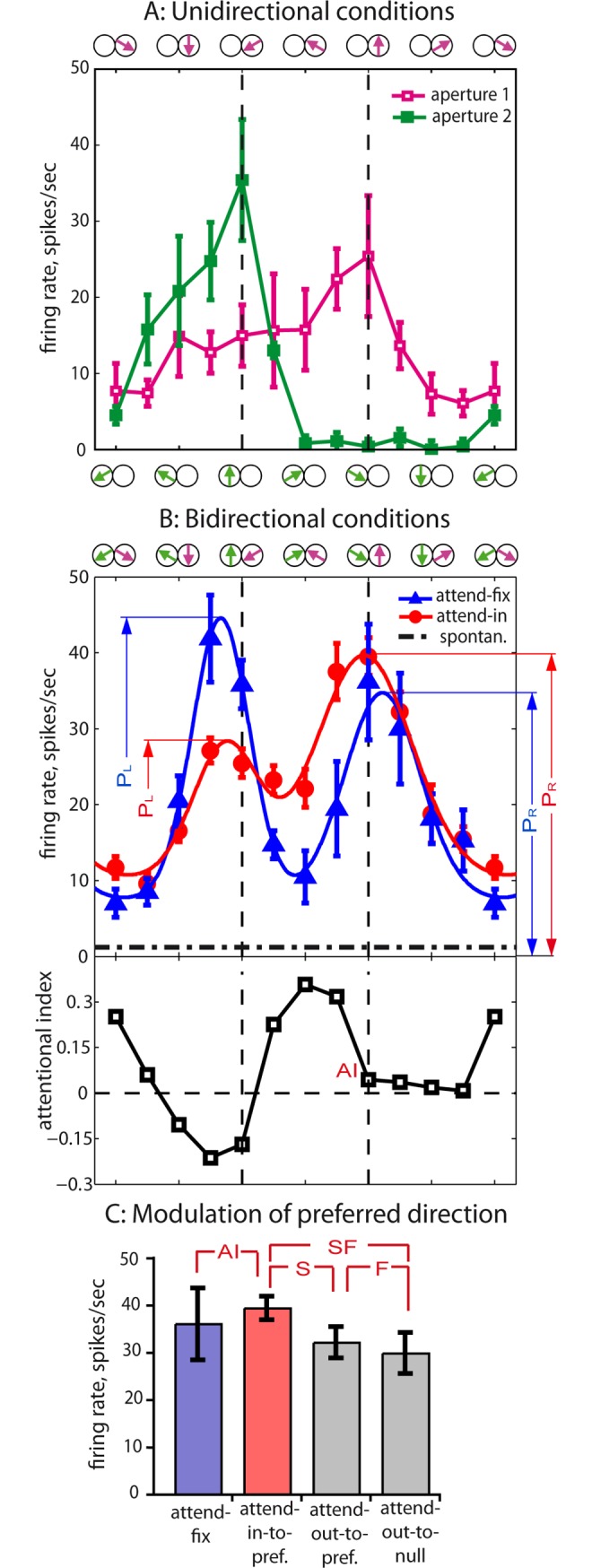
Responses of an example neuron H073-01+01. (A) Tuning to unidirectional stimuli presented in each of 2 patterns in the RF when attending to the fixation spot. The average was taken across 5 to 7 trials, error bars show 1 SEM. Motion directions of the respective pattern are depicted separately along the top and bottom x-axes. The upward arrow represents the stimulus direction that was closest to the neuron’s preferred direction. The color of the curves corresponds to the color of the arrows indicating a position of the respective pattern. Data points for the right pattern are marked by open squares, these for the left pattern by filled squares. Note that the tuning curves determined at the 2 different spatial positions within the RF were similar but not equal. (B) Bidirectional responses and their modulation by attention. The upper plot depicts tuning curves of responses to combined stimuli, plotting the average firing rates (across 5 to 12 trials) in the conditions attend-fix (blue triangles) and attend-in (red circles). Direction combinations in the RF are depicted along the x-axis by purple and green arrows corresponding to those in panel A. In the attend-in condition, the target always was the right RDP. The solid curves of respective colors represent the 2-Gaussian fits of the data (see Eq 4). Both curves show 2 peaks close to configurations when 1 of the 2 patterns moved in the neuron’s preferred direction (marked by vertical dashed lines). The height of the right and the left peaks predicted by the 2-Gaussian fits for the 2 conditions is denoted by P_R_ and P_L_ in the respective color. The average spontaneous firing across 6 trials without RDPs (attention to the fixation spot) is indicated by the horizontal dot-dashed line. The lower panel plots the attentional modulation (attend-in versus attend-fix, Eq 1) of the firing rates depicted in the upper panel. Note that strongly modulated conditions in this example don’t include the one with preferred direction in the RF (labeled by red “AI”). (C) Modulation of responses to the preferred direction in the RF in the conditions attend-fix, attend-in-to-preferred, attend-out-to-preferred, and attend-out-to-null. Comparison of the first 2 provides the AI labeled by red color in lower plot B. Comparing the latter 3 firing rates, the effects of spatial and FBA as well as their combination (indicated by the letters S, F, and SF, respectively) can be estimated. Error bars depict SEM across trials of the respective condition. For this neuron, we found on average a 22% enhancement by spatial attention (attend-in-to-preferred versus attend-out-to-preferred); FBA (attend-out-to-preferred versus attend-out-to-null) caused a 7% enhancement. The combined effect of spatial and FBA (attend-in-to-preferred versus attend-out-to-null) was 32%. The underlying data can be found in S1 Data. AI, attentional index; FBA, feature-based attention; RDP, random dot pattern; RF, receptive field.

In order to investigate how attention affects the responses of MT neuronal populations to complex (bidirectional) RDPs, we compared responses to physically identical stimuli in the RFs of 113 single MT neurons when the animals directed their attention either to the fixation spot or one of the moving patterns. For each neuron, we determined tuning curves in the bidirectional attend-fix and attend-in conditions (see Fig 1) while keeping a constant angle of 120° between the 2 RDPs in the RF. In addition to those tuning curves, we also recorded a number of control conditions to confirm that each pattern of the combined stimulus displayed alone elicits a tuned response (i.e., both patterns fall inside the classical RF) as well as to disentangle the effects of spatial and FBA. See Table A in S1 Text for the list of all conditions. Responses of an example neuron are presented in Fig 2. Here and throughout, the preferred direction is symbolized by an upward arrow, the antipreferred (null) direction, respectively, by a downward one. Fig 2A shows the average firing rates of the example cell as a function of the single pattern direction displayed in either of 2 pattern positions. In these conditions, the animals attended a luminance change of the fixation spot. The purple and green curves correspond to directions of the right and the left patterns depicted along the upper and the lower x-axis, respectively. Note that the single stimuli are simple (unidirectional), therefore the purple and the green tuning curves are bell-shaped. We combined the simple patterns so that the direction of motion in the left one was always 120° clockwise relative to that in the right one (see Materials and methods); therefore the 2 curves show peaks approximately 120° apart.

Panel B depicts the neuron’s tuning to combined stimuli, i.e., when the corresponding motion pattern are shown simultaneously (those directions are depicted along the x-axis). Both tuning curves are responses to bidirectional conditions but with different targets of attention. The attend-fix curve (blue) shows 2 peaks for the 2 stimulus configurations in which 1 of the 2 patterns moved close to the neuron’s preferred direction. This matches the results of Treue and colleagues [17] for overlapping (transparent) bidirectional patterns.

In our attend-in condition (red curve, Fig 2B), the target was always the right pattern. Though stimulation was physically identical, the cell’s responses in the 2 conditions clearly differed. We quantified attentional modulation by the attentional index (AI, Eq 1) and depicted it across the stimuli configurations in the lower plot of panel B. In this example, shifting attention from the fixation spot to a pattern in the RF caused a marginal enhancement of the peak corresponding to the stimulus when the right pattern moved in the preferred direction and a substantial suppression of the peak corresponding to the stimulus containing the preferred direction in the left (unattended) pattern. The strongest enhancement took place along the left flank of the attended peak. This important peculiarity of our data in general is discussed below.

Note that our way of plotting provides alignment of the peaks of those 2 curves with the peak locations in the unidirectional conditions (see also Fig 2A), because our bidirectional stimulation is a superposition of the single stimuli. Note also that, when attention is switched from the fixation spot to a moving RDP inside the RF, both the attended location and the attended feature change. In order to separate spatial and feature-based effects of attention [6], we compared responses to the bidirectional pattern in the RF (with aperture 1 always moving in the preferred direction) in the attend-in condition with those in the 2 attend-out conditions, i.e., with attention on the preferred or antipreferred direction, respectively. See Fig 2C and its caption for details.

The same analysis was repeated for all 113 neurons. To quantitatively estimate the effects of attention on the population responses, we fitted the response profiles in the attend-fix and the attend-in conditions by SGs (Eq 4; see example fits in Fig 2B and S4 Fig). Each of the 2 Gaussians is characterized by its amplitude, standard deviation, and location, and the overall bilobed curve has a baseline (asymptote), resulting in 7 parameters each for the 2 fits. To ensure the appropriateness of fitting the data with SG functions, we restricted our approach to cells in which both fitted Gaussians in the attend-fix condition had significant amplitudes (see Materials and methods). The fits of 24 of our neurons failed our inclusion criteria resulting in a subpopulation of 89 cells for subsequent analysis.

**Fig 3:**
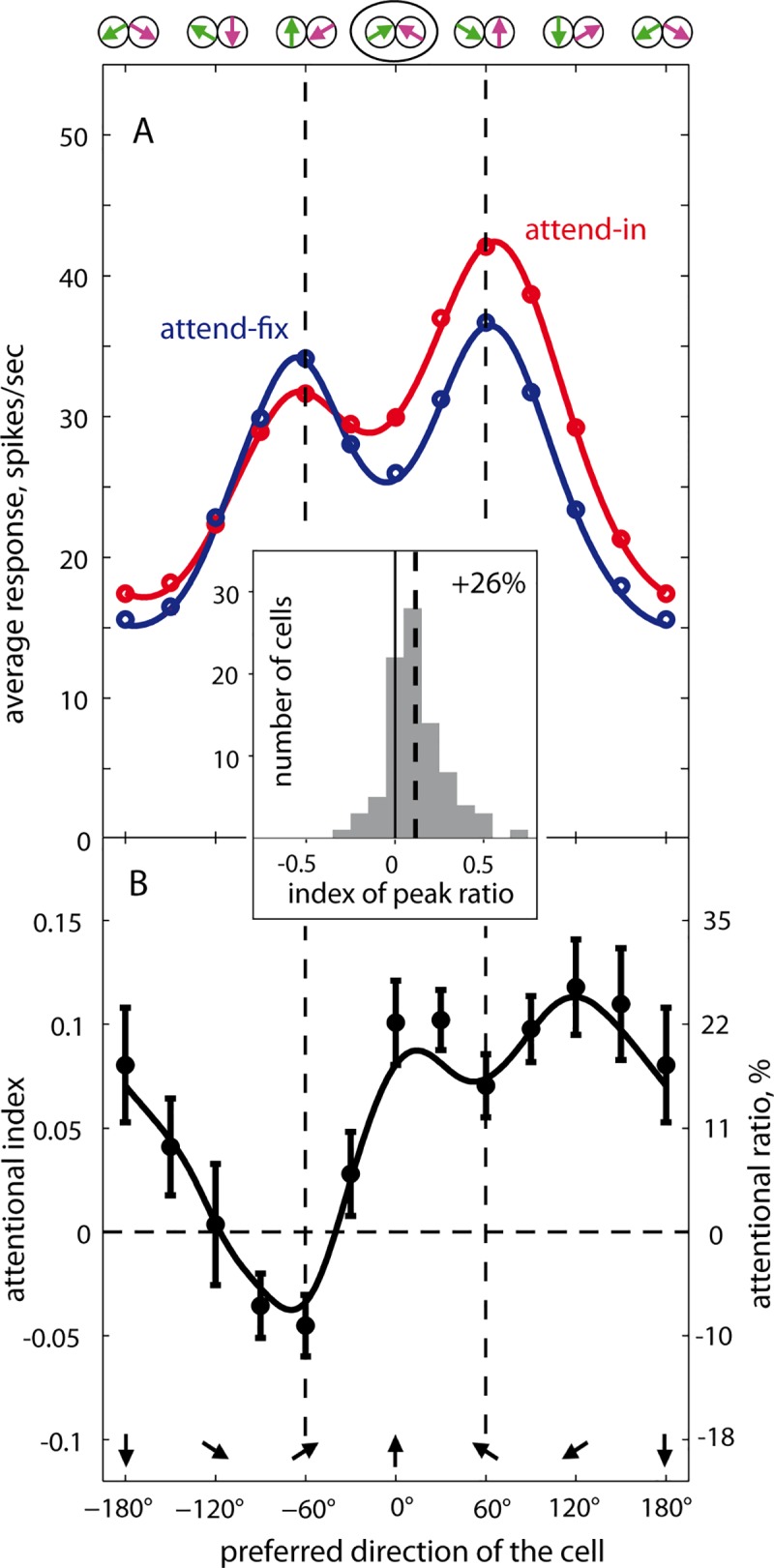
Activity profiles and response modulation across the neuronal population. The layout is similar to Fig 2B. See also S7 Fig presenting the same data differently. (A) Average responses and fits (sum of 2 Gauss functions) of the attend-fix and attend-in data from the 89 neurons included in this study. For the underlying data see S1 Data. Individual fitted tuning curves of both attentional conditions were averaged across the population. Stimulus directions relative to the neuron’s preferred (upward) direction are depicted along the upper x-axis. The curves can also be thought to represent the population response of idealized MT neurons differing only in their preferred direction (marked along the lower x-axis in panel B) to a single stimulus condition (encircled by black ellipse). The median adjusted *R*^*2*^ (Eq 5) across the cells for the attend-fix and attend-in conditions’ fits was 0.912 and 0.882, respectively. (B) Attentional modulation profile. The 12 data points are the average AIs computed across the population of neurons (see Fig 2B for AI of a single neuron; error bars are ±1 SEM). The left y-axis represents AIs, the right one shows the corresponding modulation ratios (%). The solid curve is the point-by-point modulation of the attend-in versus attend-fix population response profiles obtained by comparing the 2 fitted tuning profiles shown in panel A. (Inset) The cell-by-cell frequency distribution of AIs (attend-in versus attend-fix) for the predicted peak ratios: *AI = (PR*_*in*_ *− PR*_*fix*_*) ÷ (PR*_*in*_ *+ PR*_*fix*_*)*, where PR = *P*_*R*_ *÷ P*_*L*_ are the ratios between the heights of the right and left fitted peaks in the respective attentional condition (these values are depicted in Fig 2B). For the relative peak responses separately for the 2 conditions, see S6 Fig. Note a strong (26%) overall attentional modulation of peak responses within our neuronal population. AI, attentional index; MT, middle temporal.

Fig 3 depicts the average responses in the attend-fix and attend-in conditions across these 89 neurons (aligned according to their preferred direction) as open circles. The solid lines are the average across the individual fitted tuning curves. The response in both attentional conditions shows the double-peaked profiles already observed in the example neurons (shown in Fig 2 and S4 Fig) with the peaks representing the preferred motion of the respective pattern. Furthermore, similar to the single-neuron example, shifting attention from the fixation point into the RF enhanced the right peak representing the now-attended motion component and reduced the representation of the unattended component. The distribution of the fitted parameters across neurons is plotted in S5 Fig. The insertion in Fig 3 shows the indices distribution for the peak ratios across the 89 cells. To calculate the latter, we compared the relative height of the 2 peaks in the attend-fix and attend-in condition for each cell (for details see S6 Fig). The average modulation of 26% is well matched to the push-pull modulation effects observed in earlier studies [6,15,27,30].

Note that both the attend-fix and attend-in conditions show repulsion between the 2 peaks (though weaker than in our example, Fig 2): the average interpeak distance of 133° was significantly different from 120° (in both attentional conditions *p* < 0.005, *n* = 89, two-tailed *t* test). Furthermore, both fitted Gaussians show a reduction of their width (standard deviation) from the median of 45° in unidirectional conditions to about 35° in the attend-fix condition (*p* = 0.027, *n* = 81, Wilcoxon rank sum test; see S5 and S9 Figs).

We can interpret the response profile in Fig 3 as the population response to a single stimulus configuration (the one marked by an ellipse at the upper x-axis) of individual cells tuned to different motion directions [17]. Indeed, the single bidirectional stimulus simultaneously maximally activates 2 populations of neurons, each preferring a different direction. Assuming a homogeneous population of MT neurons, in which cells differ only by their preferred direction, the population response has the same shape as the average tuning curves depicted in Fig 3A. In other words, the neuronal tuning curves are mentally transformed into response profiles of idealized MT neurons, the RFs of which overlap the stimulus, to just the 1 bidirectional stimulus shown encircled in panel A. The preferred directions of these idealized neurons are depicted along the lower x-axis.

The attentional modulation profile is shown in Fig 3B (more details in S7–S10 Figs). For this plot, we averaged attentional indices in each stimulus condition across the population of cells. Each data point in the bottom panel is an average AI for the respective stimulus condition across all 89 cells. Every point therefore represents the attentional modulation in a subpopulation of neurons encoding the respective motion direction (black arrows). As expected, the most strongly suppressed neurons are those encoding the distractor (left pattern). Interestingly, the right peak is modulated by not only a broad enhancement of as much as 22% to 26% but also a widening of the population activity profile (for details see S2 Text and S11 Fig). This is in agreement with a recently published analysis of our data, which similarly showed a widening of the peak corresponding to the attended pattern in a majority of the recorded neurons using a model-free approach [31]. Given a midlevel of the neuronal activity (see S2 Text and S12 Fig), ceiling effects as a reason for the reduced enhancement of the preferred stimulus can be ruled out. Thus, the most strongly enhanced neurons are not the ones optimally tuned to the target pattern, rather they are those located on the flanks of the population response peak encoding the target.

Given the challenge of detecting a brief change in the target stimulus in the presence of a nearby distractor, the animals might allocate their FBA to the most informative population of neurons. A number of psychophysical and functional imaging studies [7–10] suggest that top-down modulation mainly influences the activity of those neurons, tuned to the features adjacent to the target’s features, because they are more informative for fine discriminations. We thus set out to include the idea of off-target allocation into existing models of attention. In doing so, we aim to provide a testable model that accounts for the shape of our empirical tuning curves while being more biologically plausible and having fewer free parameters than a 2-Gaussian fit.

### Models of attention: on- and off-target gain

As a starting point, we chose the conceptually simple feature-similarity gain model (FSGM; [18]), which assumes that top-down attention enhances predominantly the gain of those sensory neurons in the primate visual cortex, that are tuned to behaviorally relevant stimuli (on-target gain). In the FSGM, the amount of modulation each neuron receives is described by a monotonic function with only 2 free parameters (γ and κ) and a maximum modulation at the attended feature. Thus, the sensory response of each neuron (SG; here measured as attend-fix condition) is most enhanced by attention if the cell’s preferred feature (x) matches the attended feature (x_0_ = 60° in our case):

$$FS=SG(\gamma -\kappa \times abs{\left\|x-x_{0}\right\|}_{360}).$$ (8)

**Fig 4:**
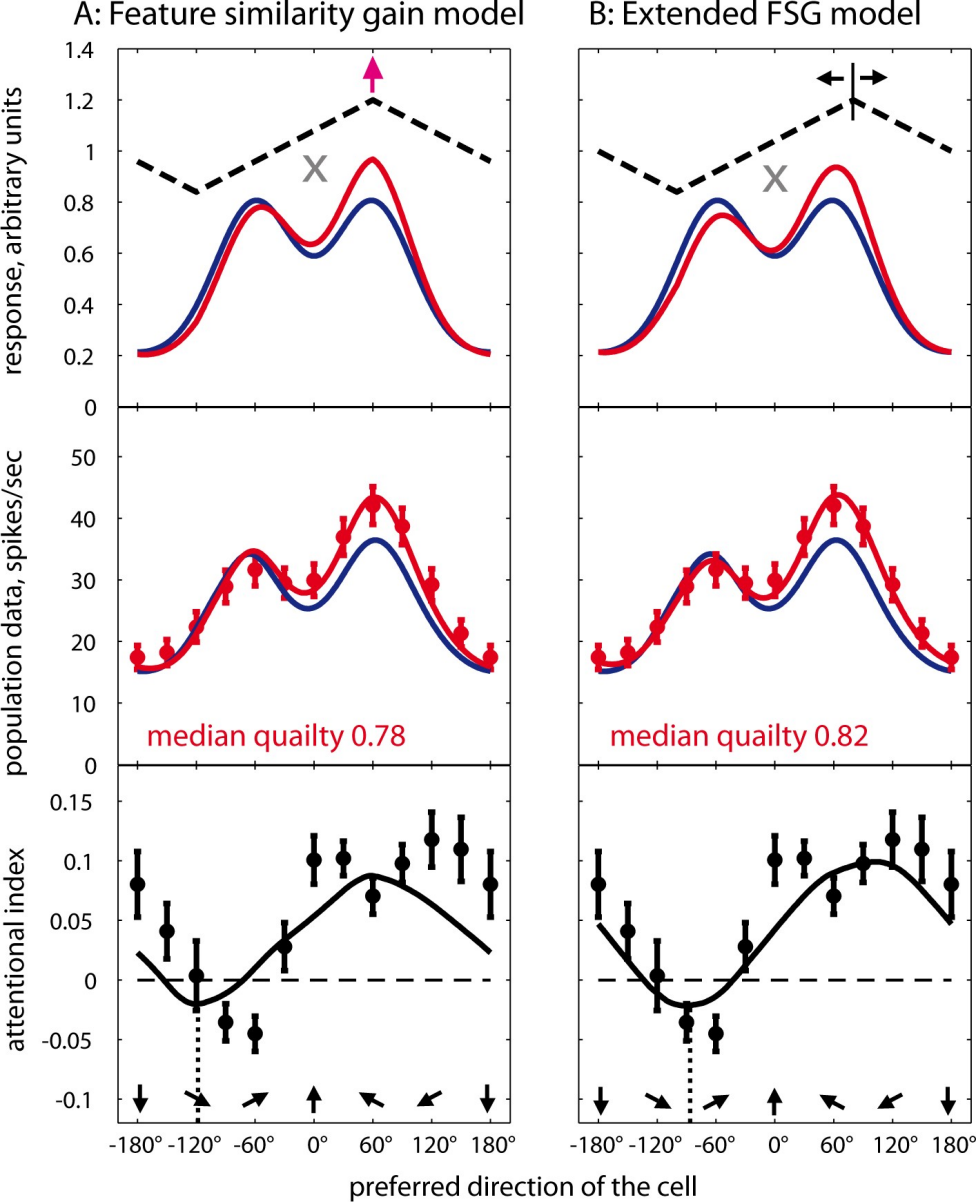
Conceptual models of attention and population fits. The upper panels represent simulations of population responses to a bidirectional stimulus moving at ±60° relative to the upward direction in attend-fix and attend-in conditions, whereas the middle and lower panels depict the respective population activity fits plotted in the same format as in Fig 3; see S1 Data for the underlying data. (A) FSGM: attend-in profile is a result of multiplication of the sensory response by a monotonic function of the similarity between the attended direction and the cell’s preferred direction (black dashed line). Fits of the attend-in data by the 2 models of attention are based on the attend-fix fit from the 2-Gaussians model; the blue curves are the same as in Fig 3. The attentional gain peaks in a subpopulation preferring the cued direction (purple arrow) and reaches its minimal value in neurons for which the cued direction is antipreferred (trough of black dashed line). (B) eFSGM. The only difference between the A and B is the location of the peak gain, which may vary in eFSGM while it is constant (on-cue) in FSGM. The median adjusted *R*^*2*^ values for both models are provided in red font. The eFSGM, despite being a relatively simple model, fits the data as well as the over-parameterized SG (*p* = 0.115, balanced one-way ANOVA on the BIC values [Eq 7] with multiple comparison of 3 models), whereas the FSGM performs significantly worse than the SG (*p* = 0.0134). BIC, Bayesian information criterion; eFSGM, extended FSGM; FSGM, feature-similarity gain model; SG, sum of 2 Gaussians.

We fit gamma and kappa for each cell individually to determine the gain profile that best transforms the attend-fix data set into the attend-in data set. In a cartoon depicting this model (Fig 4A, upper panel), the so-predicted attentional modulation is shown by the black dashed line. Unfortunately, the goodness of this model fit is not as good as the SG fit, even after adjustment to the number of free parameters (AIC, *p* < 5 × 10^−5^, two-sample *t* test).

To fully account for the complexity of combining spatial attention and FBA, we then extended the simple descriptive FSGM to include off-target attentional modulation. To this end, we introduced an additional model parameter (ξ), defining the precise focus of the animal’s FBA during each recording session and for each neuron:

$$EF=SG(\gamma -\kappa \times abs{\left\|x-\xi \right\|}_{360}).$$ (9)

This extended FSGM (eFSGM), which is derived from Eq 8, allows for attentional gain to be deployed independently of the actual stimulus input, as depicted in the upper panel of Fig 4B. The value ξ is the actually attended feature, or the focus of FBA. If ξ = x_0,_ i.e., for on-target attention, the eFSGM and the FSGM are identical. Otherwise (ξ <> x_0_), i.e., for off-target attention, there is a negative or positive offset of the peak gain from the target feature.

**Fig 5:**
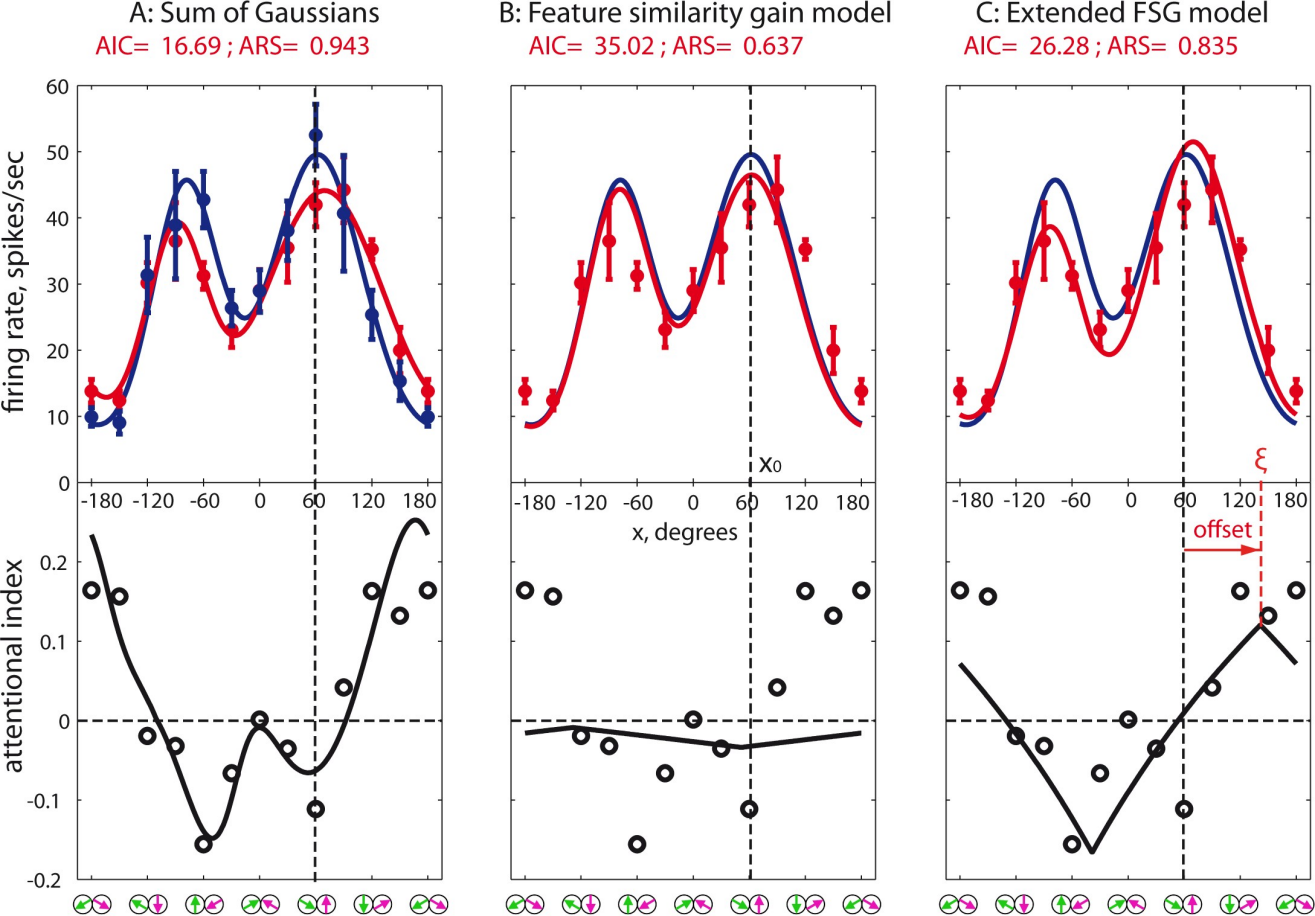
Example tuning curves of a single neuron fitted by the SG, FSGM, and eFSGM. A single-cell example (C032-02+01) of attentional gain boosting the outer flank of the target peak. The layout of all panels is similar to that of Fig 2B. (A) Responses to bidirectional stimuli in the attend-fix and attend-in conditions: data points with error bars (1 SEM) and fits by the SG (Eq 4). The lower AI plot contains data points and the solid curve calculated (Eq 1) using the measured responses and fits from the upper plot, respectively. Note that this fit just describes the data but is not based on any conceptual model. (B) Fit by the FSGM; see Eq 8. The attend-fix fit from panel A (blue curve) is multiplied by a 2-free-parameters function with a maximum at the target feature (x_0_, vertical black dashed line) to fit the red data points. Note that this model in this case completely fails to reproduce the attentional modulation. (C) Fitting the red data points by the extended FSGM (Eq 9). The blue curve is multiplied by a linear function like in panel B but having a variable position of the maximum (ξ, red dashed line) different from the target feature x_0_ by an offset (shown by red arrow). The fits quality (AIC and adjusted R^2^ values; see Eqs 5 and 6) is provided on top of the respective panels. The underlying data can be found in S1 Data. AI, attentional index; eFSGM, extended FSGM; FSGM, feature-similarity gain model; SG, sum of Gaussians.

To determine whether including the attended feature as a free parameter would improve the model fits, we fitted the attend-in responses of each neuron with the eFSGM. Fig 5B provides an example of inappropriate fit by the simple FSGM and a substantial improvement of the fit due to the included parameter.

The averaged fit across 89 neurons is depicted together with the respective AI values in Fig 4B. The AI curve peaked at about 102° (i.e., 42° away from the cued target direction, toward a greater angle). The strongest suppression was at about −90°, closely matching the data. This eFSGM fit was significantly better than the one by the FSGM, for all of our 3 criteria (adjusted R^2^: *p* < 0.0005, Wilcoxon sign rank test; AIC: *p* = 0.0003, paired 2-tailed *t* test; BIC: *p* = 0.0044, paired 2-tailed *t* test; distributions of FSGM and eFSGM goodness-of-fits: S13 Fig). This indicates that the additional parameter of the eFSGM captures a critical aspect of FBA, namely, the presence of off-target attentional allocation.

In a supplementary analysis, we also determined if the flexibility afforded by even more model parameters (including the spatial and feature extent of attention) of the normalization model of attention (NMoA; [32–37]) might provide an even better account for our observations (see Supplementary Methods for further details). The best fit of our population profiles by the NMoA is presented in S14A Fig and is comparable to the FSGM fit yet requires a large array of assumptions about the properties of the neural population at hand. However, the NMoA [37,38] can also be parameterized to account for de-aligned feature attention by adding 1 free parameter to the model. Matching the success of the eFSGM, this off-target attention also improves the prediction of the NMoA (see S2 Text and S14 Fig, compare B versus A). Here, the optimal feature-based focus is estimated to about 35° away from the cued motion (i.e., similar to the prediction of the eFSGM).

Next, we tested whether the successful inclusion of off-target attentional modulation would also be reflected in the animals’ behavior. To this end, we restricted our analysis to 74 cells with strongly significant slopes κ and reasonable estimates of angle ξ (see S2 Text). For those cells, the correlation between the attentional modulation of the target Gaussian and the focus of attention derived from the eFSGM fits (see S2 Text and S15 Fig) confirms that the observed widening of the target peak in the attend-in condition can at least partially be accounted for by a de-alignment of the attentional focus from the target feature. On the other hand, the single-cell examples (Figs 2 and 5) indicate a strongly asymmetrical effect of enhancing one flank of the target peak more than the opposite flank. Indeed, a negative attentional de-alignment would enhance the left (inner) flank of the target peak, like in the example on Fig 2, whereas a positive de-alignment boosts the right (outer) flank; see Fig 5.

**Fig 6:**
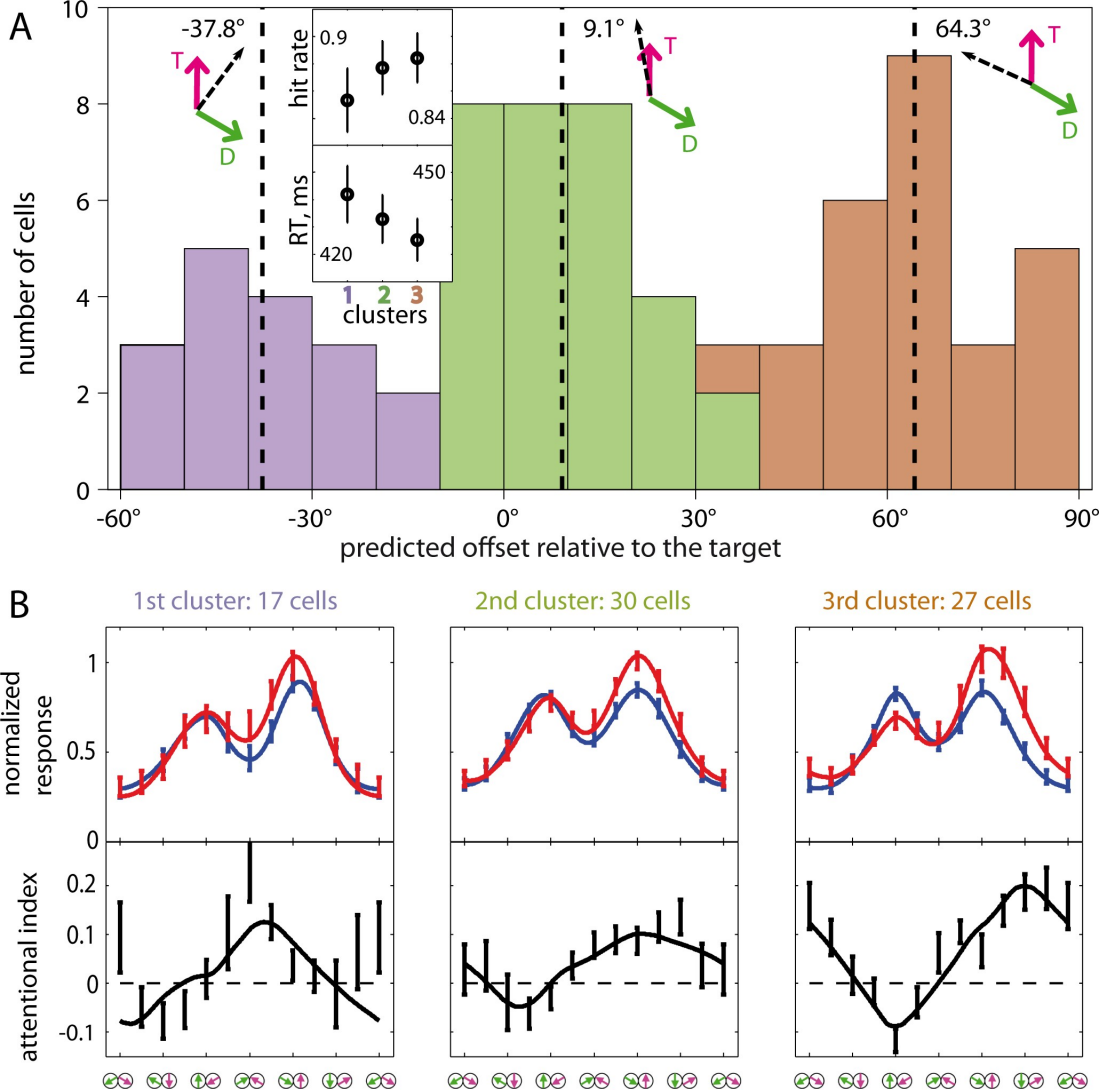
Cells clustering according to the alignment of FBA relative to the target feature estimated from the eFSGM fits. (A) Frequency histogram depicting the data from 74 cells (of both animals). For every cell, the attentional offset was computed as a difference between the predicted peak of attentional gain and the neuron’s preferred direction. It is obvious that the data fall into 3 clusters, distinguished by the bar colors. The cluster centroids are marked by dashed vertical lines with the respective values on top of the histogram. The vector cartoons show the centroid direction relative to that of the target and the distractor (marked by letters T and D respectively)T and the D. (Inset) Behavioral correlate of the 3 clusters. Mean HRs and RTs are depicted together with error bars (1 SEM). Both parameters show a trend of improvement with alignments further away from the distractor. (B) The averaged within clusters activity profiles fitted by the eFSGM depicted in 3 columns under the respective clusters of panel A. The layout is same as of Fig 3, but the upper panels here depict average responses normalized across cells to the highest attend-fix data point of each cell. Note very different modulation effects between the clusters emphasized by the plots of attentional indices. The underlying data can be found in S1 Data.; eFSGM, extended FSGM; FBA, feature-based attention; HR, hit rate; RT, reaction time.

Apart from that, there are also neurons in which the highest attentional gain is approximately aligned with the neurons’ preferred feature (on-target gain). We thus hypothesized that the recorded population may include 3 subpopulations distinguished by the direction and amount of the gain offset. To confirm the existence of distinct clusters of attentional foci, *K*-means clustering (*K* = 3, squared Euclidean distances) was performed on the angles ξ from the single neuronal data fits by Eq 9. Fig 6A presents the distribution of cells across the 3 clusters. Each cluster includes cells of both monkeys in a comparable proportion.

We hypothesized that the differences between the clusters do not reflect differences in the cells’ response properties but rather reflect distinct behavioral strategies of the animal in the respective recording sessions. We tested this hypothesis in 2 ways. First, we estimated the animals’ behavioral performance in the attend-in condition across the 3 clusters. The inset of Fig 6A shows average hit rates (HRs, ratio of correct trials excluding fixation errors) and reaction times (RTs; interval between the target change and the lever release). Although the differences between the clusters with either of the 2 measures are below significance, there is a trend of the performance improvement from the 1st to the 3rd cluster, i.e., with an increase of the attentional de-alignment in the positive direction (away from the distractor). The highest performance correlates with the positive offsets 20° to 55° relative to the target (see S16 Fig).

Furthermore, we compared the FBA foci of cells simultaneously recorded during our experimental sessions. Among the 74 neurons, 13 neuronal pairs were recorded simultaneously. Neurons of 7 pairs fell within the same clusters, 6 pairs belonged to neighboring clusters (1–2 or 2–3), and none of the pairs spanned between the first and the third clusters. A Monte-Carlo simulation of the shuffled pairs (see S17 Fig) showed that this distribution is significantly different from chance, with a bias towards similarity of the pairs of neurons in their location of the peak gain. This observation thus provides additional evidence towards a behavioral origin of the focus of FBA values.

In order to describe the typical response profiles for each of the clusters, we calculated averages across the tuning curves within the clusters. These respective cluster profiles are plotted in 3 columns of Fig 6B. The attend-fix tuning curves of neurons were fitted by the SG, whereas the attend-in ones were fitted by the eFSGM and averaged within each cluster. Overall the eFSGM provided reasonable fits for all 3 clusters. As in Fig 4, average attentional modulation was calculated based on the attend-in versus attend-fix profiles of each cluster (see lower panels of B). The plots show very different modulation effects between the clusters.

The most numerous, middle (green) cluster represents on-target gain and includes 30 cells. The distribution of the FBA foci is broad because the repulsion effect varies across the neurons. These neurons show moderate and smooth attentional modulation (with insignificant target peak widening), which is well accounted for both by the eFSGM and the FSGM.

The second most numerous (orange) cluster represents positive values of de-alignment of the focus of FBA and includes 27 cells. These cells show the most prominent target peak widening (*p* < 0.005, two-tailed *t* test) due to attentional enhancement of the outer flank (see the example in Fig 5). This group is distinguished also by the strongest suppression (19%) when the preferred direction was unattended. Including the “focus of FBA” parameter into the model resulted in a significant improvement of the fit quality in terms of the adjusted *R*^*2*^ (*p* < 0.01, Wilcoxon sign rank test).

The least numerous (blue) cluster represents negative values of de-alignment of the focus of FBA and includes 17 cells. These neurons show a strong repulsion of their response peaks in the attend-fix condition (relative to the unidirectional ones) and attraction of their peaks in the attend-in condition. Therefore this is the only group with a highly significant attentional modulation of interpeak distance (*p* = 0.0002, two-tailed *t* test). The left (inner) flank of the target peak is enhanced, just like in the example (Fig 2). The majority of the cells in this neuronal cluster show a more complex gain modulation than the linear function of the feature (see Eq 9). This resulted in less optimal fits compared to the other 2 clusters.

## Discussion

In this study, we determined how attention selectively modulates the population responses to 2 close-by stimuli in order to optimize the discriminability of the target stimulus. Both the target and the distractor were presented in the RF of individual units in motion-selective, extrastriate area MT of 2 macaque monkeys. Besides their position, the 2 stimuli differed only by a constant direction difference of 120°. Population response profiles were estimated based on the tuning curves of individual neurons, similar to previous approaches in various visual areas [17,39,40]. In short, tuning curves of individual neurons are determined by serially presenting one neuron with various stimuli differing in their direction relative to the neuron’s preferred direction. Such tuning curves also represent the response to a single stimulus by a population of idealized neurons differing in their preferred direction and therefore in their response to the given stimulus. Applying this mental transformation, the x-axis of such a response profile plot represents all MT neurons whose RFs overlap the stimulus, sorted along the x-axis by their preferred direction.

Given their relative directional separation (120°), as expected [17], the 2 RDPs induce 2 peaks in the response profile. Our data show that attending to one of the patterns enhances its representation and suppresses the representation of the unattended pattern. This well-known push-pull effect [6,15,27,30] creates a difference between the heights of the 2 peaks, i.e., between the 2 stimulus representations, of 26% on average (insert of Fig 3), representing the neural correlate of an enhanced representation of the attended stimulus at the expense of the unattended stimulus. Surprisingly, beyond this on-target gain increase, we observed a widening of the attended peak (see Fig 3 and S2 Text).

Our analysis shows that the widening of the attended peak reflects a direction-dependent attentional modulation that is not centered on the target direction but rather on directions along the slope of the response peak (off-target gain). This fits decoding schemes that focus on the locations of the steepest slope of tuned responses [41–43]. The flanks, rather than the peak, of the population response profile are the most informative about the exact identity (here the direction) of a stimulus. Thus our data of an enhanced attentional modulation along the flanks of the peak representing the attended stimulus support a highly optimized FBA allocation aimed at boosting the population’s information content [44].

One-third of all recording sessions showed a greater enhancement for the flank of the target peak facing away from the second stimulus, almost a quarter of the recordings showed a stronger effect for the flank facing the distractor (Fig 6), and for the remainder of the cells, the attentional focus appears to be centered on the target direction. We argue that these 3 types of effects are not reflecting 3 types of neurons but rather 3 behavioral strategies. This is based on our observation that simultaneously recorded pairs of neurons are more similar in their attentional focus location than expected by chance (see S17 Fig). Our data reveal an attentional system that—to implement a chosen behavioral strategy—specifically distributes attentional gain modulations along a population of neurons to enhance the response of the most task-relevant (not necessarily the most responsive) neuronal subpopulation.

Presumably the animal switches the behavioral strategy based on factors such as motivation, arousal, stimulus eccentricity, size, speed, and/or task difficulty. We have not observed any systematic variation in the gain offset or behavior depending on how far the sessions are separated in time, neither do we see any trend over the entire duration of our study.

Our physiological observations are highly reminiscent of a corresponding perceptual effect observed in human psychophysical and imaging studies based on fine feature discriminations [7,9,10]. Note that our perceptual paradigm, although often called a “change detection” task, has the characteristics of a discrimination rather than a detection task. This is because the relevant distinction between detection and discrimination tasks is the identity of the most informative neuronal subpopulation [42]. In a detection task, performance is limited by detecting a stimulus in noise, and, therefore, the most informative neuronal subpopulation is the one most sensitive to the stimulus. On-target attention ensures the best signal-to-noise ratio under such conditions [45], and correspondingly, no reshaping of the population response profiles (other than a gain change) are expected. In a discrimination task, on the other hand, the stimulus is salient but the determination of its exact identity, such as the exact direction of motion of a stimulus needs to be optimized. The most informative subpopulations for this lie along the slope of the population activity, because these neurons show the biggest change in activity when discriminating (in our task, between the direction of motion before and after a change).

Therefore the optimal gain strategy for a task-specific allocation of FBA proposed by Navalpakkam and Itti [7] for human discrimination tasks similarly applies to our experimental paradigm. The optimal gain theory argues for off-target gains and can be considered as an extension of the FSGM for predicting the distribution of gains when the target features are task-relevant. Our data thus provide the neural correlate for the perceptual observation and the implementation of the optimal gain strategy.

The de-alignment of the focus of FBA we observed is also reminiscent of a similar ability in the allocation of spatial attention. Here, directing the “spotlight of attention” not to the center of a RF but to adjacent positions has been shown to shift RF centers [46–48]. A computational approach, such as the recurrent network model of Compte and Wang [49], might help to elucidate mechanisms of highly focused attentional gain deployment in the feature domain.

Our population activity profiles are the response to the combination of the 2 motion components in the RF. Our data confirm previous observations [16] that the population response is well-approximated by the scaled sum of a neuron’s responses to the single motion patterns, except for an interaction that creates a small shift, increasing the separation between the 2 activity peaks. This quasilinear response behavior raises the conceptual question whether the attentional “saliency boost” for the attended stimulus must reflect an attentional system, such as the biased competition model [50,51], that acts at the level of the neural representations of individual stimuli, even when they fall within the same RF and are encoded by a common spike train. An alternative that does not require a selective modulation of separate stimulus representations is an attentional system that simply modulates the overall response strength of individual neurons, as hypothesized by the feature-similarity model of attention [18]. The fact that our data are well explained by a simple extension of the FSGM indicated that a differential modulation of the contribution of an attended and an unattended stimulus in the same RF to the overall response of a neuron can be achieved by a general mechanism that is stimulus independent. A computational implementation of such a mechanism was provided by an influential coupled-ring model [52] composed of a reciprocally connected loop of 2 (sensory and working memory) neuronal networks.

In addition to the feature-similarity-gain principle, we evaluated the ability of normalization models of attention by Reynolds and Heeger [37] and others, e.g. [38], to account for our observations. Our analysis showed that an NMoA with a free parameter related to the attentional de-alignment can provide a good account of the data.

Our results are also compatible with a probability-mixing model based on the Neural Theory of Visual Attention [53]. The latter predicts that neurons in extrastriate visual cortex encode the presence of more than one distinct stimulus in their RF by alternating between response states, each predominantly representing one of those stimuli. This hypothesis was recently tested [54] using our data set. Evidence in support of such a multiplexing behavior was found by analyzing spike trains of individual trials rather than average responses across trials. The distribution of attentional gain modulations along the population we report here was replicated by Li and colleagues [54] by adjusting the multiplicative perceptual bias of neurons encoding a given feature.

In summary, our study documents the neural basis of a sophisticated attentional system that is able to differentially distribute FBA, such that the strongest attentional modulation is directed toward those neurons, which are the most informative contributors to the task at hand. Such a system represents an optimal adaptation of primate vision to the challenges of a complex and highly cluttered environment.

## Supporting information

S1 Data Main data. Excel spreadsheet containing, in separate sheets, the underlying numerical data and statistical analysis for Figs 2, 3 (3 individual: individual numerical values summarized in Fig 3), 4, 5, 6A and 6B, as well as S15–S17 (in Fig 6A sheet).
(XLSX)

S2 Data Supporting data. Excel spreadsheet containing, in separate sheets, the underlying numerical data and statistical analysis for S3, S4, S5, S6, S7, S8, S9, S10, S11, S12, S13 and S14 Figs.
(XLSX)

S1 Text Supporting methods. (PDF)

S2 Text Supporting results. (PDF)

S1 Fig Mapping of the RF and tuning properties estimation. (A) Stimulus arrangement. A triangular lattice of up to 13 probe locations was centered on the manually estimated location of the RF “hotspot” (shown by a cross). The trial started with fixation of a light gray spot on a dark gray background (the colors are shown inversed). Each trial contained an alternating motion stimulus (RDP) at one of the probe locations. The motion speeds and directions were randomly drawn in intervals of 250 ms from 3 speeds and 12 directions. Example set of directions and speeds of a probe stimulus within 1 trial is depicted by color arrows. The monkey was rewarded for detecting a brief (130 ms) luminance decrease in the fixation spot. (B) Example of tuning curves. Three direction-tuning curves were constructed for each of the locations. Tuning at different speeds (4, 8, and 16°/sec) is shown, respectively, in light green, green, and dark green. The small black circle in the central plot depicts the spontaneous firing rate. The example cell was particularly selective for patterns moving at 8°/sec (the largest ratio of responses to the preferred and antipreferred directions at most of the probes locations); the preferred direction was about 240°. Positioning of the apertures for the main task is shown by 2 magenta circles. The latter were larger than the probes (4° in diameter) and covered an area with robust and similar tuning. RDP, random dot pattern; RF, receptive field.
(PDF)

S2 Fig Example of spike density functions for the neuron H089-01+02, conditions with the preferred motion in the RF (see Materials and methods). Time is in miliseconds relative to the cue onset. The analysis time window is marked in yellow. RF, receptive field.
(PDF)

S3 Fig Distribution of numbers of repeated recordings per condition in the population of 89 neurons. The frequencies represent all bidirectional conditions and all neurons. Median value for attend-fix is 4 repetitions, for the attend-in and–out: 7 repetitions.
(PDF)

S4 Fig Example fit of neuron C047-03+01 tuning curves by the SG. Layout is similar to Fig 2. The average firing rates in the attend-fix (blue triangles) and the attend-in (red circles) conditions are shown together with error bars (1 SEM). The SG fits are shown by solid lines of the respective color. The inserted table shows the fitting parameters with 95% confidence bounds as well as goodness of fit (adjusted R^2^). SG, sum of Gaussians.
(PDF)

S5 Fig Distribution of the fitted parameters (SG) in the subpopulation of 89 neurons included in Fig 3. Binning is according to the absolute values of the parameters. The ordinate represents the number of cells in each bin. Parameters of the attend-fix condition fits are depicted in blue color, those of the attend-in condition in red. Parameter values provided on top of each histogram correspond to Eq 4. They are presented in the form: <parameter> = <mean value>; sem = <standard error of mean>; med = <median value>. c_1_-c_2_ (in degrees) is interpeak distance between the 2 Gaussian components. See S2 Data for the parameters numerical values. SG, sum of Gaussians.
(PDF)

S6 Fig Analysis of the relative predicted peak responses in the subpopulation of 89 neurons. Distribution of the indexes between the fitted peak firing rates was calculated for each attentional condition separately: (A) attend-fix; (B) attend-in. Indexes of the right versus left peak firing rates were calculated for each individual tuning curve. We used an equation similar to those described in Materials and methods: Index = (P_R_ − P_L_) ÷ (P_R_ + P_L_), where P_R_ and P_L_ are, respectively, the height of the right and the left peaks predicted by the Gaussian model. On average, the peaks showed no significant difference in the attend-fix condition, whereas in the attend-in condition the peak corresponding to the attended pattern was significantly higher than the other one.
(PDF)

S7 Fig Normalized activity profiles and response modulation in the population of 89 neurons included in in Fig 3. Upper panel shows average response profiles of the attend-fix and attend-in data. Firing rates of each neuron were normalized by the highest response point of the attend-fix condition; error bars represent ±1 SEM. The lower panel depicts individual data points of attentional indices of each neuron (Eq 1) by gray “+” (without outliers) as well as averaged AI across the population (black line). Circles mark same data points as in Fig 3B. AI, attentional index.
(PDF)

S8 Fig Tuning curves and AIs plotted separately for the cell populations of 2 monkeys. Layout is similar to the one used in Fig 3. The AI curve of monkey H compared with that of monkey C showed a stronger enhancement in most of the stimulus configurations. On the other hand, the suppression effect in monkey C was highly significant, whereas monkey H showed on average no suppression. Note though a large variability of the modulation effect in both monkeys when the direction of the attended pattern was close to antipreferred. As it was suggested by Khayat and colleagues (*J Neurosci*. 2010), the modulation in such configurations may be caused by FBA differentially modulating the strength of direction-selective inputs carrying signals from the 2 patterns into the recorded neurons. The AI was on average near zero in both monkeys when the antipreferred pattern is attended and the other pattern moves 120° apart (third point from the left), exactly the same as one observed by Khayat and colleagues for a similar configuration (see their Fig 7C). AI, attentional index; FBA, feature-based attention.
(PDF)

S9 Fig Population response to bidirectional and unidirectional patterns: 2 animals, C and H, 88 cells (46 and 42, respectively). This subpopulation strongly overlaps with the 1 of 89 neurons depicted in Fig 3. The layout of the upper panel is similar to Fig 2A and 2B but combines all 4 average tuning curves in 1 plot. Fitting of each of unidirectional responses by single Gaussians shows that the tuning widths are very close to 45° (in terms of parameter b of Eq 3, median across cells). Comparison of the unidirectional and bidirectional (attend-fix) profiles show, in general, a quasilinear summation of the 2 component responses with some nonlinear interactions (like repulsion, width reduction, and unequal weighting of the peaks). The lower panel depicts the modulation profile, attend-in versus attend-fix (only bidirectional conditions) with error bars representing ±1 SEM taken across the 88 cells. As in the other subpopulations, the AI curve shows a trough at a point where the preferred direction is attended (compare with Fig 3, S8 Fig and S10A Fig). AI, attentional index.
(PDF)

S10 Fig Population response: 2 animals (C and H), 113 cells (46 and 67, respectively). (A) Normalized response profile (upper panel) and modulation profile, attend-in versus attend-fix (lower panel). The layout is similar to Fig 3. Neuronal responses for a given neuron were normalized to the highest firing rate in the attend-fix bidirectional condition in that neuron and aligned to the preferred direction. Error bars represent ±1 SEM taken across all cells. Modulation ratios for the conditions with the preferred stimulus in the RF are given in blue, the peak modulation ratios in red. The insertion shows mean ranks of modulation indexes (attend-in versus attend-fix) across 5 stimulus conditions close to the “preferred direction attended.” The conditions were compared by nonparametric Friedman's test with a follow-up multiple comparison test (see sheet S10 in S2 Data). Error bars denote 95% confidence intervals of the estimated mean ranks. The testing shows that 2 conditions (marked by red error bars with the respective *p*-values) when attention was somewhat away from the preferred direction have mean ranks significantly different from the condition in which just the preferred direction was attended (marked by blue error bars and dotted lines). The median goodness of the fits (adjusted R^2^ values) for the 113 accepted cells was 0.91. (B–D) Attentional modulation observed when the preferred direction was presented in the RF across the complete data set. The histograms show distribution of response changes caused by spatial (B), feature-based (C), and combined (spatial+feature, D) attention, respectively. Binning of the x-axis is according to the AIs. Bars include 2 differently colored stacks for the data from the 2 animals: brown for monkey C and gray for monkey H. On average spatial attention modulated responses by 16% (*p* < 0.0001, paired two-tailed *t* test), which is in agreement with earlier reports: Treue and Martinez Trujillo (*Nature*, 1999): 12%; Katzner, Busse and Treue (*Front Syst Neurosci*. 2009): 18%. The average modulation by FBA is 6% (*p* < 0.01), somewhat smaller than in the studies mentioned above (13% and 12%, respectively). A possible reasoning is provided in the main text. Means of the indexes are marked by the vertical dashed lines (red for monkey C, black for monkey H). Throughout, neuronal responses in monkey C were modulated less strongly than those in monkey H. Generally, the population of recorded neurons shows significant modulation by all 3 forms of attention (except for feature-based effects in the monkey C). Note that all 3 assessments of different forms of attention are based on the responses to individual stimulus constellations rather than the overall population activity. AI, attentional index; FBA, feature-beased attention; RF, receptive field.
(PDF)

S11 Fig Frequency histograms of relationship (computed as attentional indices) between the fitted data in attend-in versus attend-fix condition across the population of 89 neurons. The individual histograms plot the attentional modulation of the amplitudes of the 2 Gauss functions (A, B), widths (standard deviations) of the 2 Gauss functions (C, D), distances between the centers of the 2 Gauss functions (E), asymptotic values of the fits in the 2 conditions (F). Histograms B and D denote the parameters of the component Gaussian corresponding to the target pattern, histograms A and C denote those of the distractor pattern. Mean indices are marked by vertical dashed lines and given in the insertions to the histograms together with the respective *p*-values (paired *t* test). (G, H) Relationship between the fitted peak firing rates in attend-in versus attend-fix conditions across the population of 89 neurons. Note that, unlike panels A and B, both of these histograms compare values relevant to both Gaussian components. In this case, the right peak gain (H) and the left peak gain (G) show the modulation ratios of, respectively, +14% and −10%, which are close to those estimated by our point-by-point responses averaging.
(PDF)

S12 Fig Correlation between the saturation index and widening index of the attended component in a subpopulation of 70 neurons. The peak response saturation was calculated for each cell as a ratio of maxima across 6 points: attend-in responses to preferred-60°… preferred+90° (aperture 1) in the numerator; 3+3 peak responses in the 2 attend-fix unidirectional conditions (preferred ±30° in aperture 1 or 2) in the denominator. The results show no correlation (r = 0.038; *p* = 0.756).
(PDF)

S13 Fig Distributions of the goodness-of-fits for the population of 89 neurons, eFSGM versus FSGM estimated by 3 criteria. (A) adjusted R^2^, (B) AIC, and (C) BIC; for their definitions, see Materials and methods, and see S2 Data for the numerical values. The diagonal red line in each plot depicts points of equal goodness. Vertical and horizontal dashed lines depict the median (in A) or the mean (in B and C) of the criterion across cells. Performance of the 2 models was compared by Wilcoxon sign rank test across adjusted R^2^ values and by paired two-sided *t* test across AIC or BIC values; the calculated *p*-values are displayed on the respective plots. All 3 comparisons show a significant difference between the prediction qualities by the 2 models in favor of the eFSGM, accounting for de-aligned feature attention. AIC, Akaike information criterion; BIC, Bayesian information criterion; eFSGM, extended FSGM; FSGM, feature-similarity gain model.
(PDF)

S14 Fig Fitting the NMoA to the averaged response profiles (89 neurons). Both fits assume a modulated baseline. Further assumptions: (A) attention is focused on the target; (B) attention may focus off-target. In the latter case, therefore, there is an additional free parameter of the FBA defocus. Note an increased goodness (adjusted R^2^) of 0.958 in the model B compared with that of 0.931 in A. Because we used the mean firing rates (blue and red circles) across neurons rather than fitting each neuron individually, the goodness of fit is not directly comparable to that of the model fits presented in Fig 4. See S2 Text for further explanations. The NMoA, like the extended FSGM, provides significantly better fits if the defocus of FBA is allowed. FBA, feature-based attention; FSGM, feature-similarity gain model; NMoA, normalization model of attention.
(PDF)

S15 Fig Distribution of the target peak widening indices against the predicted by Eq 9 focus of FBA. Seventy-four cells with reliably predicted values are included. (A) Second-order polynomial fit to this distribution is depicted by dashed curve. This fit includes a significant square coefficient (considering the 95% confidence interval) and accounts for about 25% of the data variance (r^2^ = 0.247). (B) Scatter plot representing the target peak widening indices as a function of the adjusted focus of FBA values. The latter consider variation of tuning width across neurons and the peaks repulsion effect. Here, the second-order polynomial fit accounts for 35% of the data variance. See S2 Text for further explanation. FBA, feature-based attention.
(PDF)

S16 Fig Correlation between the predicted by Eq 9 off-target focusing of attention and behavioral accuracy of the monkey during the recording session (attend-in condition). Upper panel depicts HR (ratio of correct trials excluding fixation errors); middle panel depicts RT, an interval (ms) between the target change and the lever release as functions of distance between the attentional focus and the target feature. Open circles denote the measured (fitted) points (for the values, see sheet Fig 6A in S1 Data); red dashed curves are locally weighted linear regressions to smooth the data. The 2 measures show the highest performance when the highest attention gain peaked at about 30° to 35° off-target, away from the distractor direction. The HR curve shows the second maximum at a negative angle (about 25° off-target in the direction of distractor). Lower panel plots IE (solid red curve) calculated as the reaction time normalized by the hitrate: IE = RT ÷ HR (see Romei and colleagues, *Curr Biol*. 2009). The predicted offset values were binned in 4 bins (approximately 35° wide, containing similar cell numbers per bin). The lowest IE (highest performance) took place at moderate positive de-alignments (third bin, 20°–55°), which was significantly different from the bins 1 and 2 (*p* < 0.05, Kruskal-Wallis test with multiple comparisons of mean ranks). When the attended feature was close to the target or de-aligned in the direction of the distractor, the performance was highly variable and on average lower than when the offset was away from the distractor. HR, hit rate; IE, inverse efficiency; RT, reaction time.
(PDF)

S17 Fig Distribution of simultaneously recorded units (13 pairs) as belonging to same, neighboring, or distant clusters shown in Fig 6. The green dashed curve shows the actual distribution (7, 6, and 0 pairs, respectively; for the values, see sheet Fig 6A in S1 Data). The box plot presents a distribution of shuffled pairs in which the cluster numbers of the first unit in each pair were randomly permuted 1,000 times. The red mark shows the median, edges of the box show the 25th and 75th percentiles, and the whiskers extend to the most extreme data points of the latter distribution (excluding outliers which are not shown). We tested if the shuffled data are distributed with the median equal to the actual numbers. The sign-test showed highly significant deviation from the respective medians in the “same” and “distant” groups (*p* < 0.0001), whereas it did not reach significance in the “neighboring” group. Therefore, the simultaneously recorded pairs of neurons are more similar in their attentional focus location than expected by chance.
(PDF)

## Abbreviations

AI: attentional index
AIC: Akaike information criterion
AR: attentional ratio
BIC: Bayesian information criterion
eFSGM: extended FSGM
FBA: feature-based attention
FSGM: feature-similarity gain model
HR: hit rate
LAVES: Niedersaechsisches Landesamt fuer Verbraucherschutz und Lebensmittelsicherheit
MT: middle temporal
NMoA: normalization model of attention
RDP: random dot pattern
RF: receptive field
RT: reaction time
SG: sum of Gaussians
