# Supplementary material for: Strategic deployment of feature-based attentional gain in primate visual cortex
Source: PLoS Biol. 2019 Aug 6;17(8):e3000387. doi: 10.1371/journal.pbio.3000387 (PMC6684042; doi:10.1371/journal.pbio.3000387)
Supplement: S1 Text — (PDF) [file pbio.3000387.s003.pdf]

Strategic deployment of feature-based attentional gain in primate visual cortex

Vladislav Kozyrev, Mohammad Reza Daliri, Philipp Schwedhelm & Stefan Treue

PLoS Biology, 2019

Supporting Methods

Mapping of the receptive field

After isolating a single unit, its classical receptive field (RF) was identified by its response to a stationary random dot pattern (RDP) that was manually swept across the screen while the monkey performed a fixation task. The task was detection of a luminance change of the fixation spot (12x12 min of arc, luminance change from 85 to 52 cd/m^2, background luminance of 15 cd/m^2). In several trials the 'hotspot' with the most robust firing was identified.

Before the main experiment was started, we had to characterize speed and direction selectivity of the cell as well as find locations and sizes of two stationary apertures for stimuli within the RF to drive the neuron approximately equally. Therefore we mapped the RF by presenting series of moving RDPs (probes) at several positions around the estimated hotspot of the RF while the monkey went on performing the same fixation task. The round probes were presented at 7 to 13 locations on a triangular lattice, being 3° in diameter and 3° spaced from the neighbors (S1 Fig). Individual dot size was 6 min. of arc at a dot density of 10 dots per square degree. The number of included probes varied depending on the RF size and configuration. Motion speeds and directions were randomly drawn from three speeds (4, 8 and 16 °/sec) and 12 evenly spaced directions between 0° (=upwards) and 330°. Each trial contained an alternating every 250 ms pattern in one of the selected locations and included up to 23 analysis intervals. Responses to the individual speed-direction combinations were defined as mean firing rates in an interval of 50-250 ms after onset of the specific combination.

Within 50 to 100 trials we obtained 2-5 repetitions of each stimulus combination, necessary for getting direction tuning curves at three speeds in each of the selected probe locations. These tuning curves were visualized online. Based on them, the experimenter chose locations and sizes for the two stimulus apertures yielding similar tuning. The apertures might not necessarily overlap some of the probes, rather had to match the shape

of the excitatory part of the RF. For the following experiments, a speed level was chosen at which there was a clear direction tuning, the direction yielding the highest response was defined as preferred.

Conditions of the main experiment

Table A. Complete list of experimental conditions

Condition	Attention target			Stimuli in the RF		Output
	Type	Location rel. to RF	Direction of motion	Aperture 1	Aperture 2	
attend-fix, bidirectional	fixation spot	outside	—	12 directions	direction in aperture 1+120°	tuning curve
attend-in, bidirectional	RDP	inside, aperture 1	12 directions	12 directions, same as target	direction in aperture 1+120°	tuning curve
attend-fix, unidirectional (1)	fixation spot	outside	—	12 directions	—	tuning curve
attend-fix, unidirectional (2)	fixation spot	outside	—	—	12 directions	tuning curve
attend-out-to-preferred	RDP	outside	preferred	preferred	preferred+120°	single data point
attend-out-to-null	RDP	outside	preferred+ +180°	preferred	preferred+120°	single data point
spontaneous activity	fixation spot	outside	—	—	—	single data point

Data analysis

Individual tuning curves were aligned according to the neuron's preferred direction. Since the stimuli directions were calculated based on the pre-estimated preferred direction, in most of the cases no extra manipulation with the data set was required. In a number of cases though, a misalignment occurred due to errors in the preferred direction estimation

or matching the stimuli to the properties of a different channel recorded simultaneously. In those cases the complete set of tuning curves could be shifted 30° or 60° in either direction in order to get the maximal responses in the attend-fix unidirectional conditions (if measured, else - two peaks in the attend-fix bidirectional condition) aligned with the respective tuning curves of other neurons recorded in the standard situation. Data sets, which required larger shifts, were disregarded in the further analysis.

Nonlinear least square fitting method with weights was employed for quantitative estimation of modulation in the tuning curves between different attentional conditions. As the weighting factors, inverse values of standard errors at individual data points were used. The standard errors, which were smaller than $SE_{min} = 2 \div \sqrt{n-1}$, where n is number of repetitions, were corrected to SE_{min}. All calculations were performed with custom scripts written in MATLAB (versions R2007b and R2014a, The MathWorks, Natick, MA) using the Curve Fitting Toolbox.

The following parameter constraints were used in the Gaussian fits:

$a = 0 \ldots 1.2 \times (R_{max} - R_{min})$, *spikes/sec* $b = 15° \ldots 90°$

$c = 0° \ldots 120°$ $d = -50 \ldots 100$, *spikes/sec*

Distribution of the fitted parameters by the sum of two Gaussians model (attend-fix and attend-in conditions) is shown in S5 Fig, the numerical values are provided in S2 Data.
