# Supplementary material for: Strategic deployment of feature-based attentional gain in primate visual cortex
Source: PLoS Biol. 2019 Aug 6;17(8):e3000387. doi: 10.1371/journal.pbio.3000387 (PMC6684042; doi:10.1371/journal.pbio.3000387)
Supplement: S2 Text — (PDF) [file pbio.3000387.s004.pdf]

Strategic deployment of feature-based attentional gain in primate visual cortex

Vladislav Kozyrev, Mohammad Reza Daliri, Philipp Schwedhelm & Stefan Treue

PLoS Biology, 2019

Supporting Results

Sum-of-two-Gaussians fits show widening of the tuning curves

Fitting the response profiles with the sum of two Gaussians allows us to interpret the attentional effects as the independent modulation of the two Gaussian components of a given fit. In addition we can evaluate the attentional modulation of each of the three parameters of the two Gauss functions (height, width, position) independently. S11 Fig depicts the distributions and averages of the attentional indexes of these fitting parameters. Histogram A shows that attention reduced the amplitude of the distractor component of the population response on average by 22% ($p<0.001$, two-tailed t test) while the target component's amplitude was enhanced by 11% ($p = 0.013$, histogram B).

Confirming an effect visible in Fig 3, histogram D in S11 Fig shows a widening (16%, $p<0.001$) of the target peak. The distractor peak (histogram C) shows a trend of widening. Two-way ANOVA with the factors "peak" (target, distractor) and "attention" (in, fix) results in a significant widening of the both peaks ($p=0.027$), while interaction between the factors does not reach significance ($p=0.138$). Therefore, attention might modulate the width of both peaks. But we observe a stronger modulation along the peak representing the attended stimulus, furthermore, the strongest attentional enhancement takes place at its flanks rather than the peak.

Histograms E & F show on average no attentional modulation of the distance between the two peak locations (though a number of neurons substantially exhibited either the peaks' attraction or repulsion) and a trend for an enhanced baseline response (9%, $p = 0.074$).

It should be noted that the peak responses to the bidirectional stimulation are not identical to the amplitude of the underlying Gauss functions (e.g. because the two Gauss functions ride on an asymptotic value). Therefore the attentional modulation of these peak responses might be a more intuitive representation of the effects of attention. Panels G and H of S11 Fig show the corresponding histograms for the overall peak responses,

derived from our fits. They indicate that the average modulation of the population response in area MT to bidirectional patterns is a push-pull combination of a 14% enhancement ($p < 0.001$) of the population's response to the attended stimulus and a 10% suppression ($p < 0.001$) of the peak response to the unattended stimulus.

Recalling the attentional widening of the tuning profile within the target peak, we wondered whether the reduced enhancement of the preferred motion direction might be due to some sort of ceiling effect. Indeed, when attending in the RF where the optimal direction and speed are present (see Methods), the neurons may approach their attainable firing rate. Alternatively, a neuron would show a higher firing in other conditions, likely those with the preferred direction presented in the RF alone. In order to test this possibility, we correlated the target peak widening with the response saturation (considering neurons with higher firing in the attend-in relative to the unidirectional conditions as more saturated). The data show no correlation, ruling out the ceiling effect as an explanation for the observed widening of the attended peak (see S12 Fig).

Normalization model fits

We fitted our data with a modified version of the widely used normalization model of attention (NMoA, [1]). This NMoA generates a population response to a sensory stimulation in a given attentional condition by normalizing the populations' excitatory input (excitatory drive) with a 'suppressive drive', generated by convoluting the excitatory drive with suppressive filters. Attention, in the form of an attentional field, composed of the spatial and feature-based attentional state of the organism, is multiplied with the stimulus-driven input and thus modulates the excitatory drive.

$$R(x, \theta) = \frac{A(x, \theta)E(x, \theta)}{S(x, \theta) + \sigma}$$

Where $R(x, \theta)$ is the response of a neuron with its receptive field centered at x and its feature tuning centered at θ. $A(x, \theta)$ is the attentional gain for that spatial location and feature and $E(x, \theta)$ is the excitation of that neuron caused by sensory stimulation. $S(x, \theta)$ is the activity of the suppressive surround, normalizing the excitatory drive:

$$S(x, \theta) = s(x, \theta) * [A(x, \theta)E(x, \theta)]$$

Where $s(x, \theta)$ is the suppressive filter (e.g. the amount of surround suppression) and $*$ is convolution.

In order to fit this model to our data, we assumed biologically plausible parameters (see below) for our population of direction-selective neurons and fitted the population's mean responses in both conditions, attend-fix and attend-in simultaneously, since they shared all model parameters not related to the focus of attention.

We used our own implementation of the model, which assumes a circular feature-space for both the stimulation and the attention fields [2]. Therefore, in our fits, we express the width of the feature-attention spotlight in terms of von-Mises concentration parameter κ. Furthermore, to convert model responses to actually observed firing rates, we introduced a scaling parameter, which was multiplied with the population response. In order to confirm that our code produces a similar result as the original code written by Reynolds & Heeger [1], we compared the output of both versions for the set of parameters best fitting our neuronal dataset. Differences between the two versions were small with a maximal difference of less than 4 sp/s in the attend-in condition.

We used a nonlinear least-squares procedure to find parameters best explaining our observed neuronal responses, fitting always both conditions, attend-in and attend-fix simultaneously with shared free parameters. Free parameters were the concentration κ of feature-based attention (FBA), the gain factor of attention, the modulated baseline and the multiplication factor for the final conversion to spike rates.

Fixed model parameters were assumed to either match our observed values or to be biologically plausible. They were defined as follows: receptive field size (excitatory x-width): 4.25°; tuning width (excitatory θ-width): 45°, estimated by fitting with Gaussians the unidirectional tuning curves shown in S14 Fig; inhibitory x-width: 12.7°; inhibitory θ-width: 360° (extremely broad directional normalization); normalization sigma: 10^{-6}; no unmodulated baseline and a fixed attentional suppression of 0.5 at unattended locations. Spatial and feature-based attentional modulations were combined multiplicatively.

The stimulation field was defined to contain two stimuli with an arbitrary contrast of 1, centered at arbitrary spatial positions -1° and 1°, each with a size of σ=1° in space, and κ≈32.8 (corresponding to σ=10°) in feature-space. They differed apart from their spatial locations only by their motion directions of 60° and -60°, respectively.

Attention was defined as directed at the spatial location and the motion direction (or a similar direction for fits with defocused feature attention) of the target stimulus. The extent of the spatial attention spotlight was fixed to σ=5° in the attend-in condition and to σ=1° in the attend-fix condition. In the latter, spatial attention was furthermore directed at a location 10 degrees away from the two stimuli and feature-attention to motion was absent. We took the population response of all simulated units with a receptive field center at 0° (in between the two stimuli, thus receiving equal sensory input from both) as a predictor of our measured population response.

Peak widening and feature attention focus

Based on the extended feature-similarity-gain model (eFSGM) fits as well as NMoA with variable focus of attention, it is possible to explain the mentioned above widening of the right peak in the attend-in condition fits in respect to that in the attend-fix condition by the offset of feature-based attention (compare Fig.1C in [3]).

In order to quantitatively estimate the relationship between the response profiles shape modulation and location of the focus of FBA, we correlated the values of the target peak widening based on the sum-of-Gaussians fits (identical to the indices presented in S11 Fig D) and the defocusing of FBA predicted by the eFSGM (equation 9 of the main text): ξ–60° (in respect to on-target case). To ensure appropriate fits by the latter model, we restricted our approach to the neurons showing: 1) a significant slope κ (95% confidence interval should not include zero); and 2) not too vague estimate of angle ξ (95% confidence interval should not cover the whole range of its variation, namely [0; 150]). The fits of 15 neurons were thus excluded from this analysis.

Distribution of the target peak widening indices against the relative FFBA for the remaining 74 cells is presented by a scatter plot in S15 Fig A. Generally, when the FFBA is close to zero (focusing attention on the target direction), the widening on average doesn’t take place (index~0). On the other hand, widening of the target peak increases with an increase of attentional defocus in either direction. We fitted a second-order polynom to this distribution depicted by dashed curve in S15 Fig A. This fit includes a significant square coefficient (considering the 95% confidence interval) and accounts for about 25% of the data variance (r^2=0.247). Similar conclusions can be driven also from a

linear correlation between the FFBA absolute values (merging positive and negative defocus) and the peak widening indices. There we have got a significant correlation (r=0.276, p=0.0186).

Furthermore, considering variation of tuning width across neurons and the above mentioned peaks repulsion effect, we may adjust the estimate of attentional focus by correcting the latter by the target peak position (c_1) and normalizing by the target peak width (b_1) available from the sum-of-Gaussians fit of the attend-fix condition: $\tilde{\xi} = (\xi - c_1)/b_1$. Scatter plot in S15 Fig B represents the target peak widening indices as a function of those adjusted values. Dependence of the peak widening from the FFBA here is stronger than that in the panel A: the second-order polynomial fit (dashed line) accounts for more than 35% of the data variance (r^2=0.3534). It should be mentioned though, that the described adjustment refers only to parameters of the right peak, while attention obviously modulates the whole response profile.

References

1. Reynolds JH, Heeger DJ. The normalization model of attention. Neuron. 2009;61: 168–85.
2. Schwedhelm P, Krishna BS, Treue S. An extended normalization model of attention accounts for feature-based attentional enhancement of both response and coherence gain. PLoS Comput Biol. 2016;12: e1005225. doi:10.1371/journal.pcbi.1005225
3. Pouget A, Bavelier D. Paying attention to neurons with discriminating taste. Neuron. 2007;53: 473–475. doi:10.1016/j.neuron.2007.02.004
