# Supplementary figures and images for: Strategic deployment of feature-based attentional gain in primate visual cortex
Source: PLoS Biol. 2019 Aug 6;17(8):e3000387. doi: 10.1371/journal.pbio.3000387 (PMC6684042; doi:10.1371/journal.pbio.3000387)

**A**

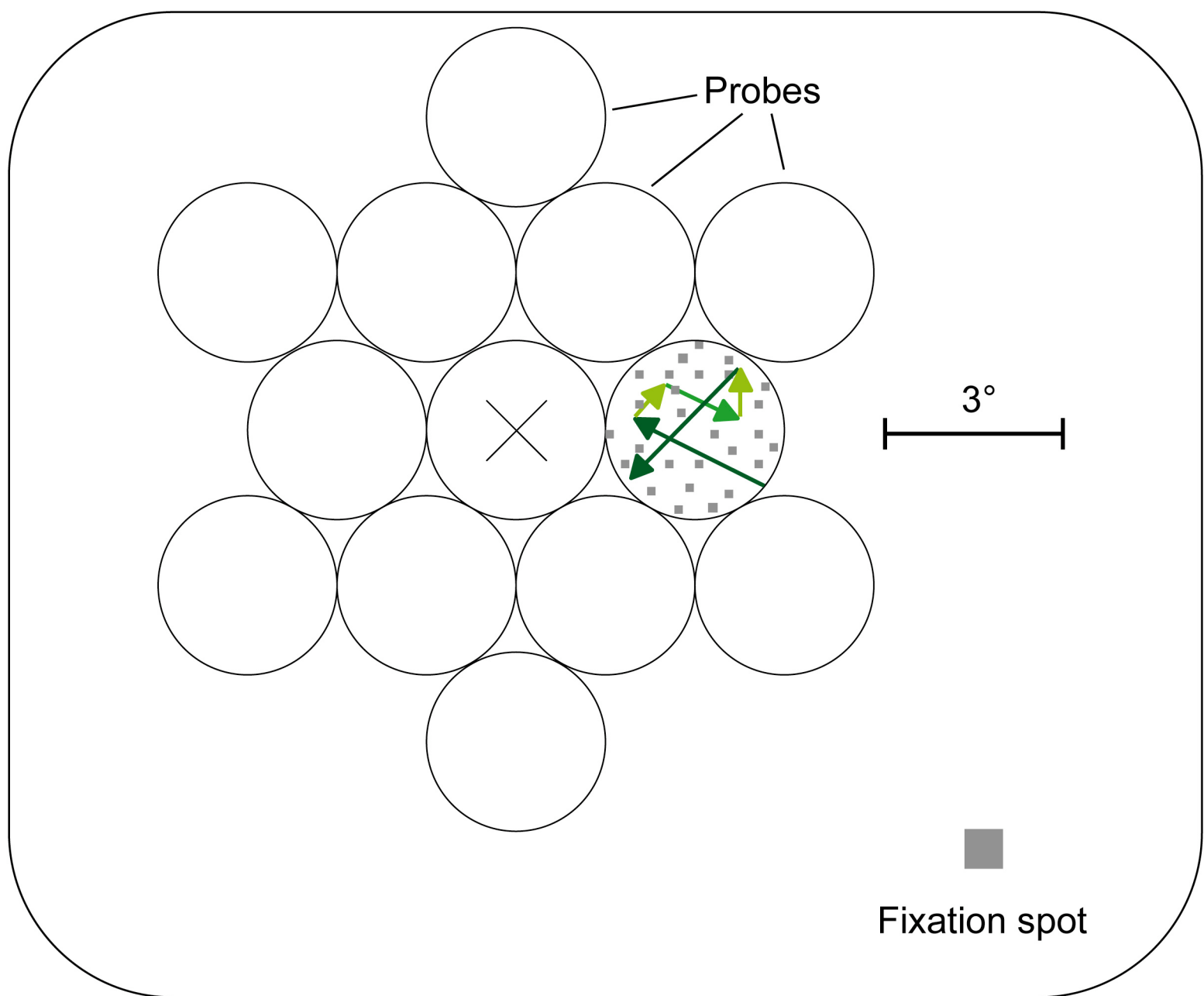

**B**

Example tunMap-H083-01+01  
channel 1

- speed 4 °/sec
- speed 8 °/sec
- speed 16 °/sec

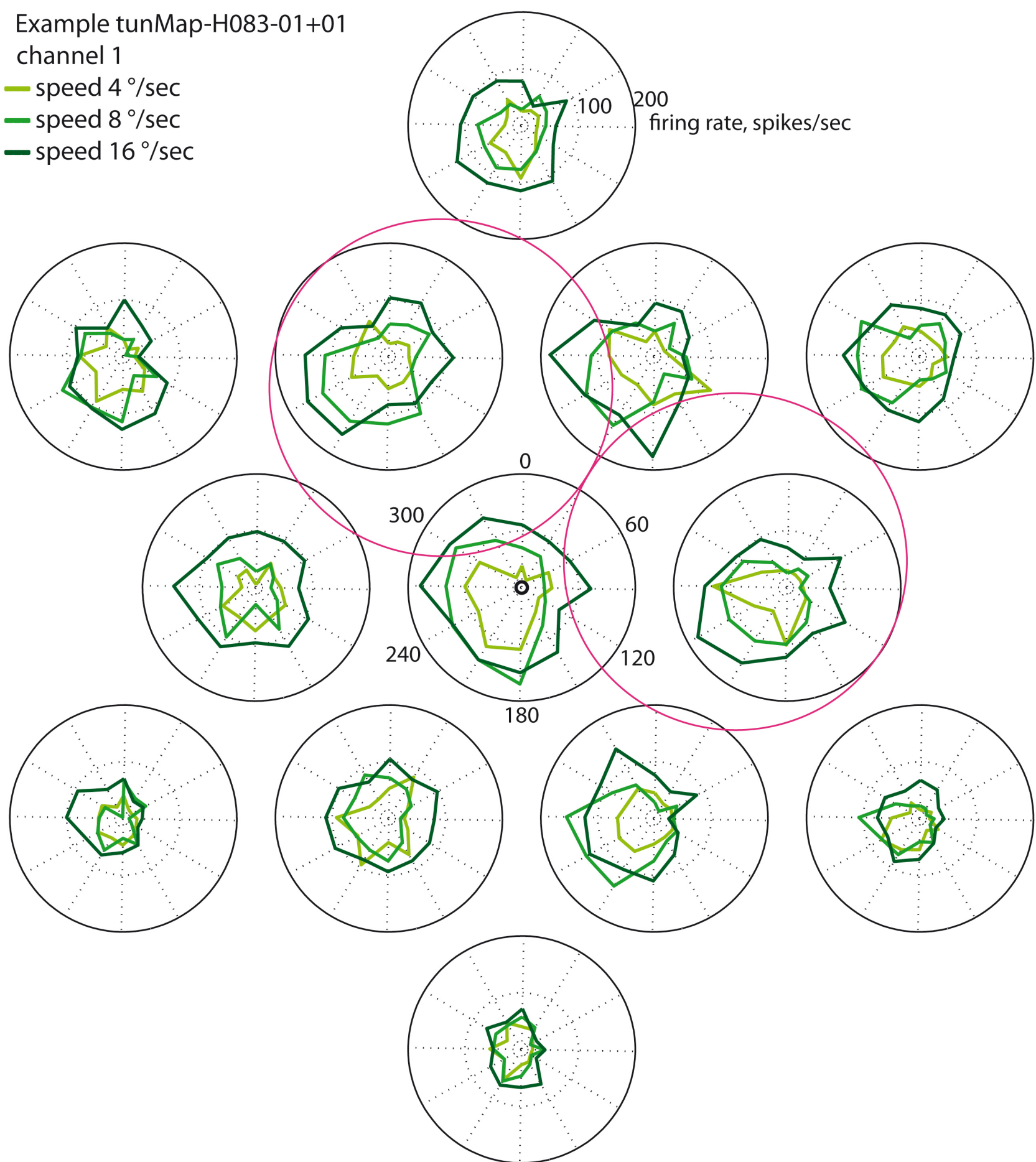

Supplement: S1 Fig — (A) Stimulus arrangement. A triangular lattice of up to 13 probe locations was centered on the manually estimated location of the RF “hotspot” (shown by a cross). The trial started with fixation of a light gray spot on a dark gray background (the colors are shown inversed). Each trial contained an alternating motion stimulus (RDP) at one of the probe locations. The motion speeds and directions were randomly drawn in intervals of 250 ms from 3 speeds and 12 directions. Example set of directions and speeds of a probe stimulus within 1 trial is depicted by color arrows. The monkey was rewarded for detecting a brief (130 ms) luminance decrease in the fixation spot. (B) Example of tuning curves. Three direction-tuning curves were constructed for each of the locations. Tuning at different speeds (4, 8, and 16°/sec) is shown, respectively, in light green, green, and dark green. The small black circle in the central plot depicts the spontaneous firing rate. The example cell was particularly selective for patterns moving at 8°/sec (the largest ratio of responses to the preferred and antipreferred directions at most of the probes locations); the preferred direction was about 240°. Positioning of the apertures for the main task is shown by 2 magenta circles. The latter were larger than the probes (4° in diameter) and covered an area with robust and similar tuning. RDP, random dot pattern; RF, receptive field. (PDF) [file pbio.3000387.s005.pdf]

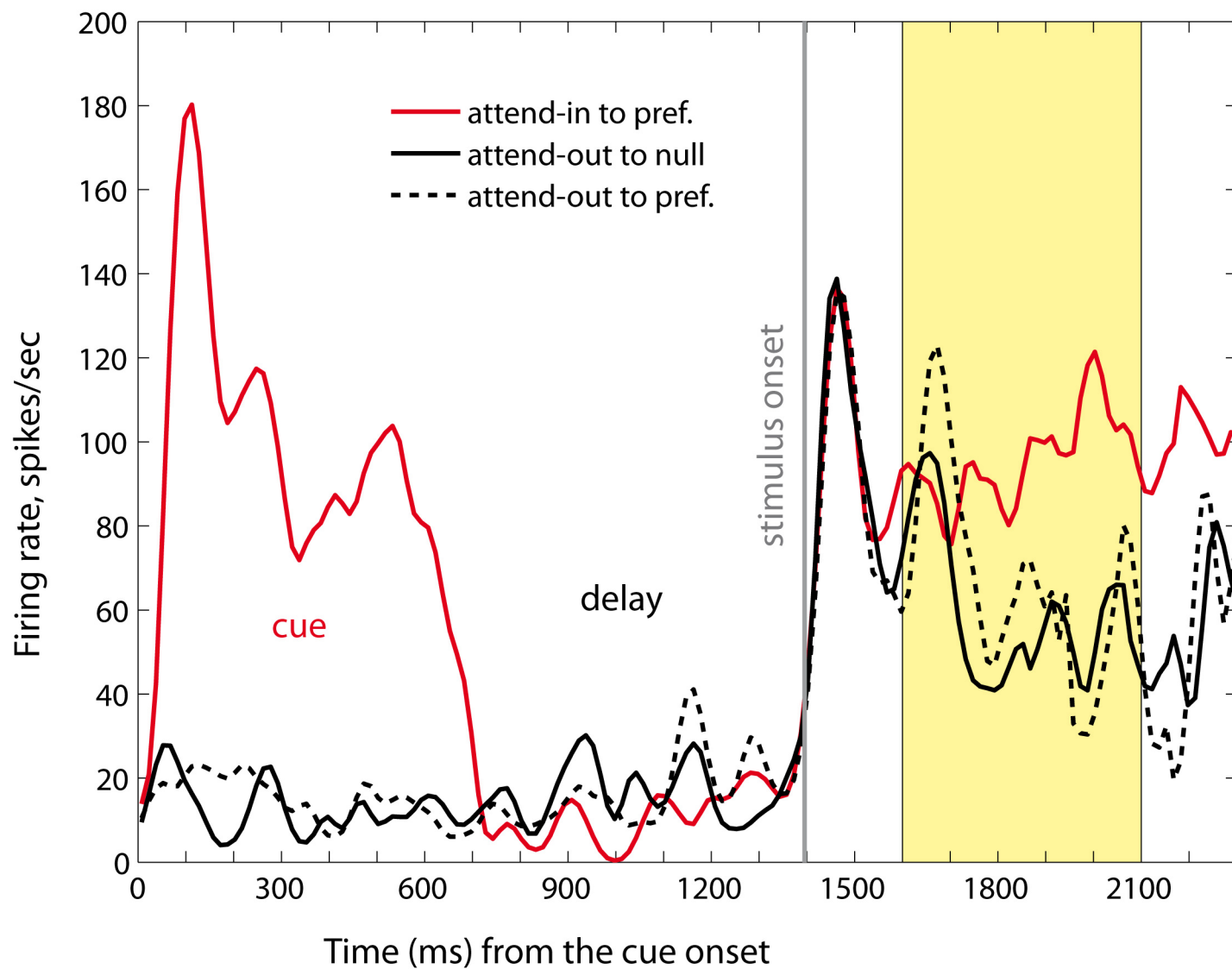

Supplement: S2 Fig — Time is in miliseconds relative to the cue onset. The analysis time window is marked in yellow. RF, receptive field. (PDF) [file pbio.3000387.s006.pdf]

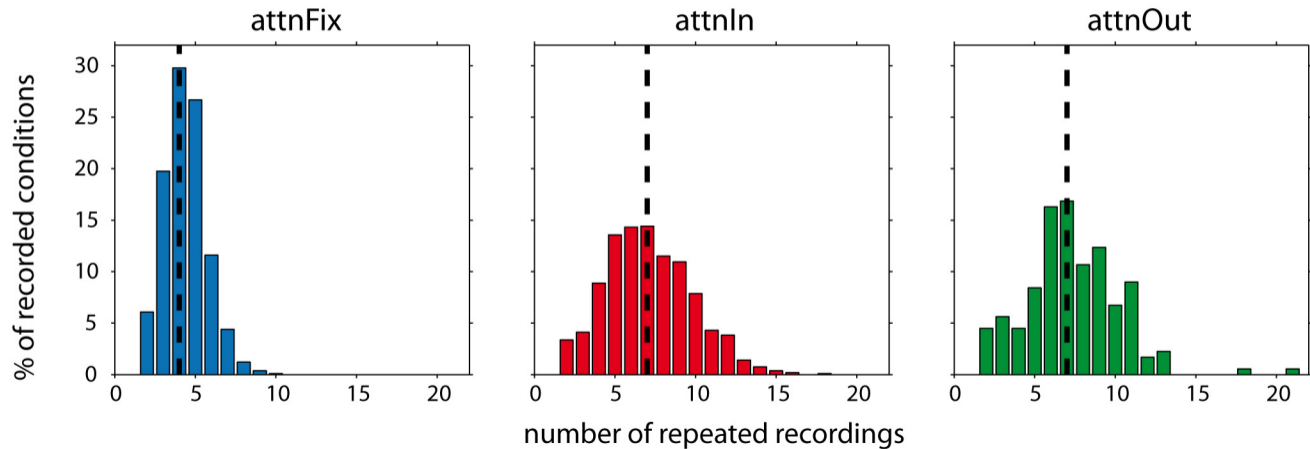

Supplement: S3 Fig — The frequencies represent all bidirectional conditions and all neurons. Median value for attend-fix is 4 repetitions, for the attend-in and–out: 7 repetitions. (PDF) [file pbio.3000387.s007.pdf]

# Example neuron C047-03+01

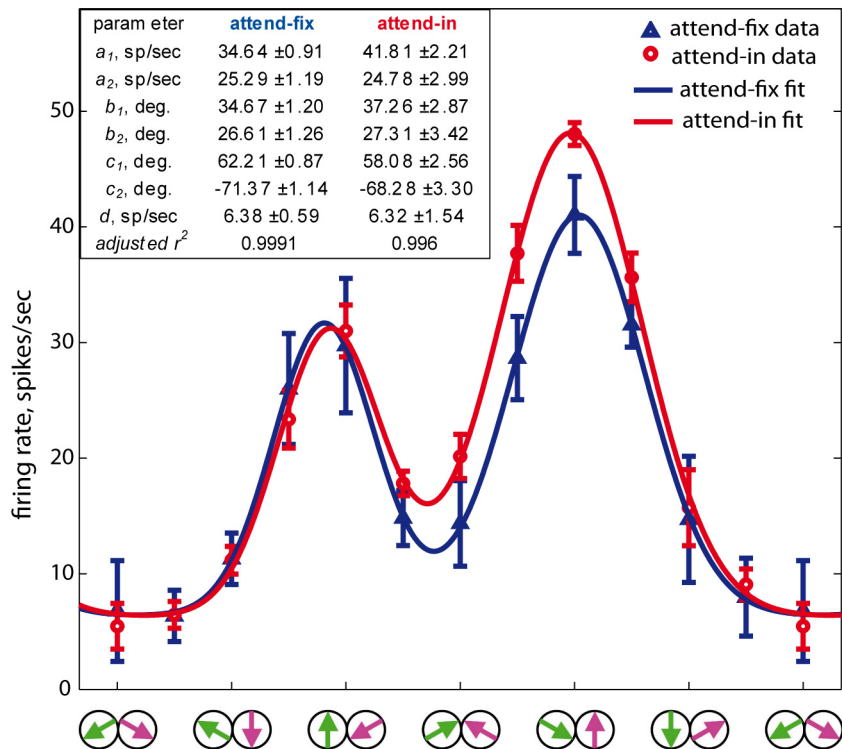

Supplement: S4 Fig — Layout is similar to Fig 2. The average firing rates in the attend-fix (blue triangles) and the attend-in (red circles) conditions are shown together with error bars (1 SEM). The SG fits are shown by solid lines of the respective color. The inserted table shows the fitting parameters with 95% confidence bounds as well as goodness of fit (adjusted R2). SG, sum of Gaussians. (PDF) [file pbio.3000387.s008.pdf]

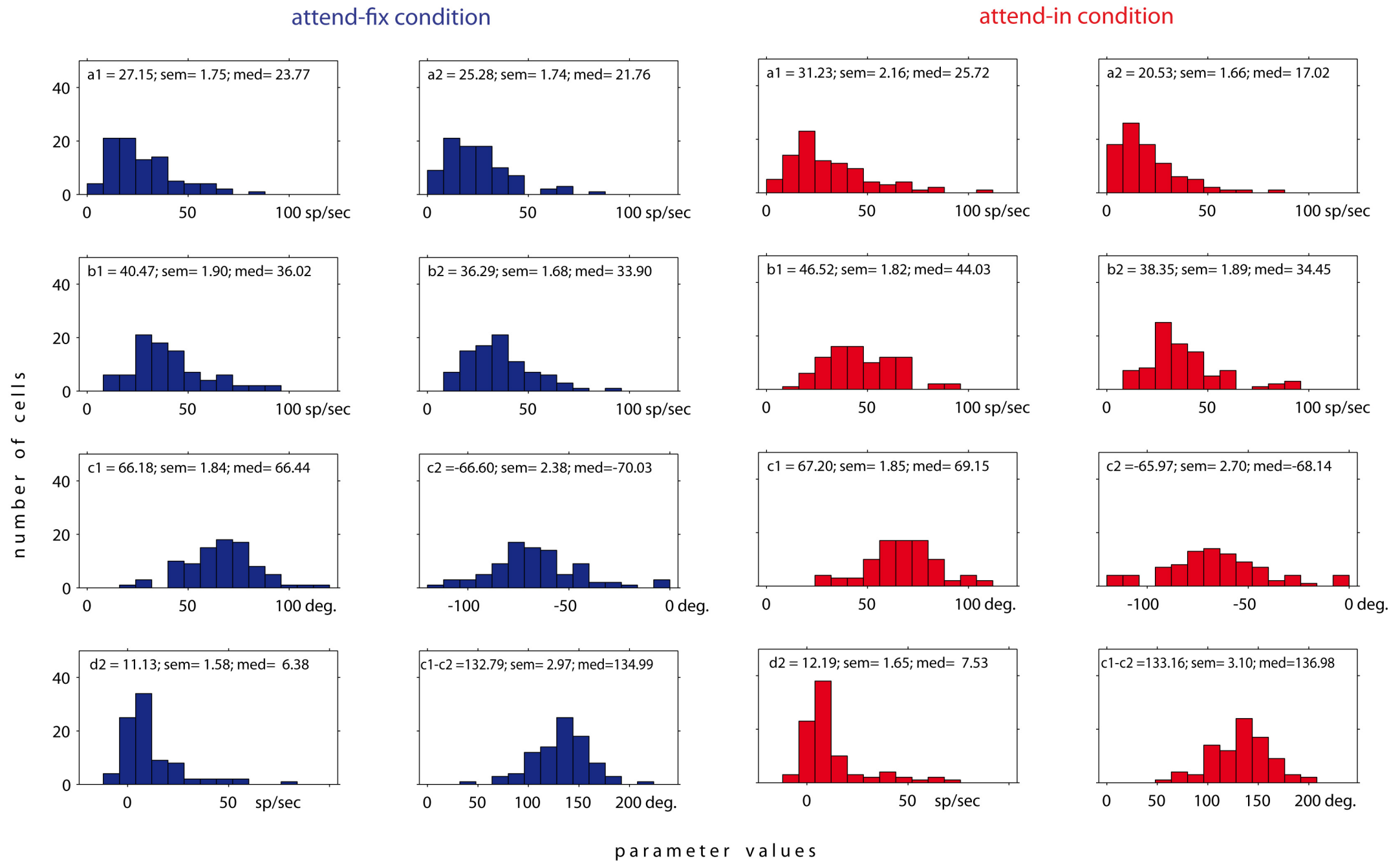

Supplement: S5 Fig — Binning is according to the absolute values of the parameters. The ordinate represents the number of cells in each bin. Parameters of the attend-fix condition fits are depicted in blue color, those of the attend-in condition in red. Parameter values provided on top of each histogram correspond to Eq 4. They are presented in the form: = ; sem = ; med = . c1-c2 (in degrees) is interpeak distance between the 2 Gaussian components. See S2 Data for the parameters numerical values. SG, sum of Gaussians. (PDF) [file pbio.3000387.s009.pdf]

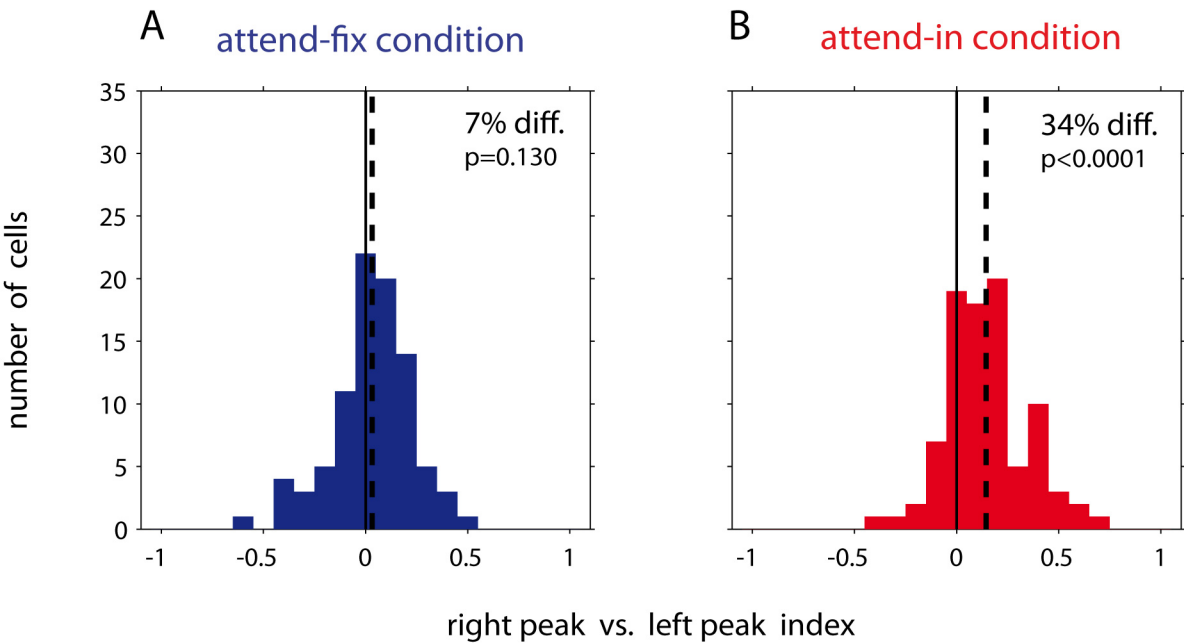

Supplement: S6 Fig — Distribution of the indexes between the fitted peak firing rates was calculated for each attentional condition separately: (A) attend-fix; (B) attend-in. Indexes of the right versus left peak firing rates were calculated for each individual tuning curve. We used an equation similar to those described in Materials and methods: Index = (PR − PL) ÷ (PR + PL), where PR and PL are, respectively, the height of the right and the left peaks predicted by the Gaussian model. On average, the peaks showed no significant difference in the attend-fix condition, whereas in the attend-in condition the peak corresponding to the attended pattern was significantly higher than the other one. (PDF) [file pbio.3000387.s010.pdf]

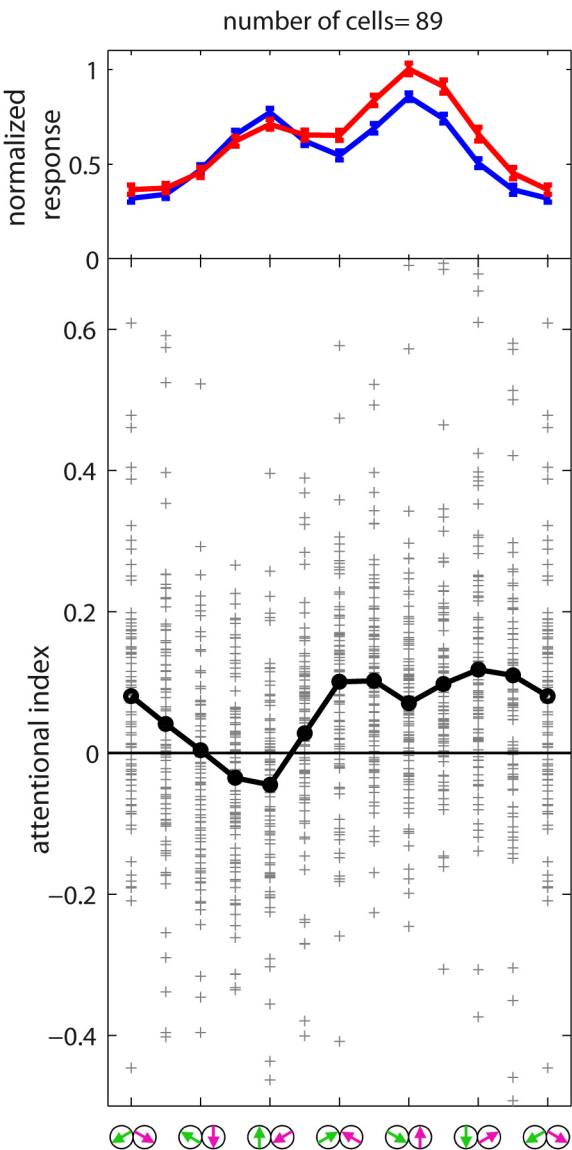

Supplement: S7 Fig — Upper panel shows average response profiles of the attend-fix and attend-in data. Firing rates of each neuron were normalized by the highest response point of the attend-fix condition; error bars represent ±1 SEM. The lower panel depicts individual data points of attentional indices of each neuron (Eq 1) by gray “+” (without outliers) as well as averaged AI across the population (black line). Circles mark same data points as in Fig 3B. AI, attentional index. (PDF) [file pbio.3000387.s011.pdf]

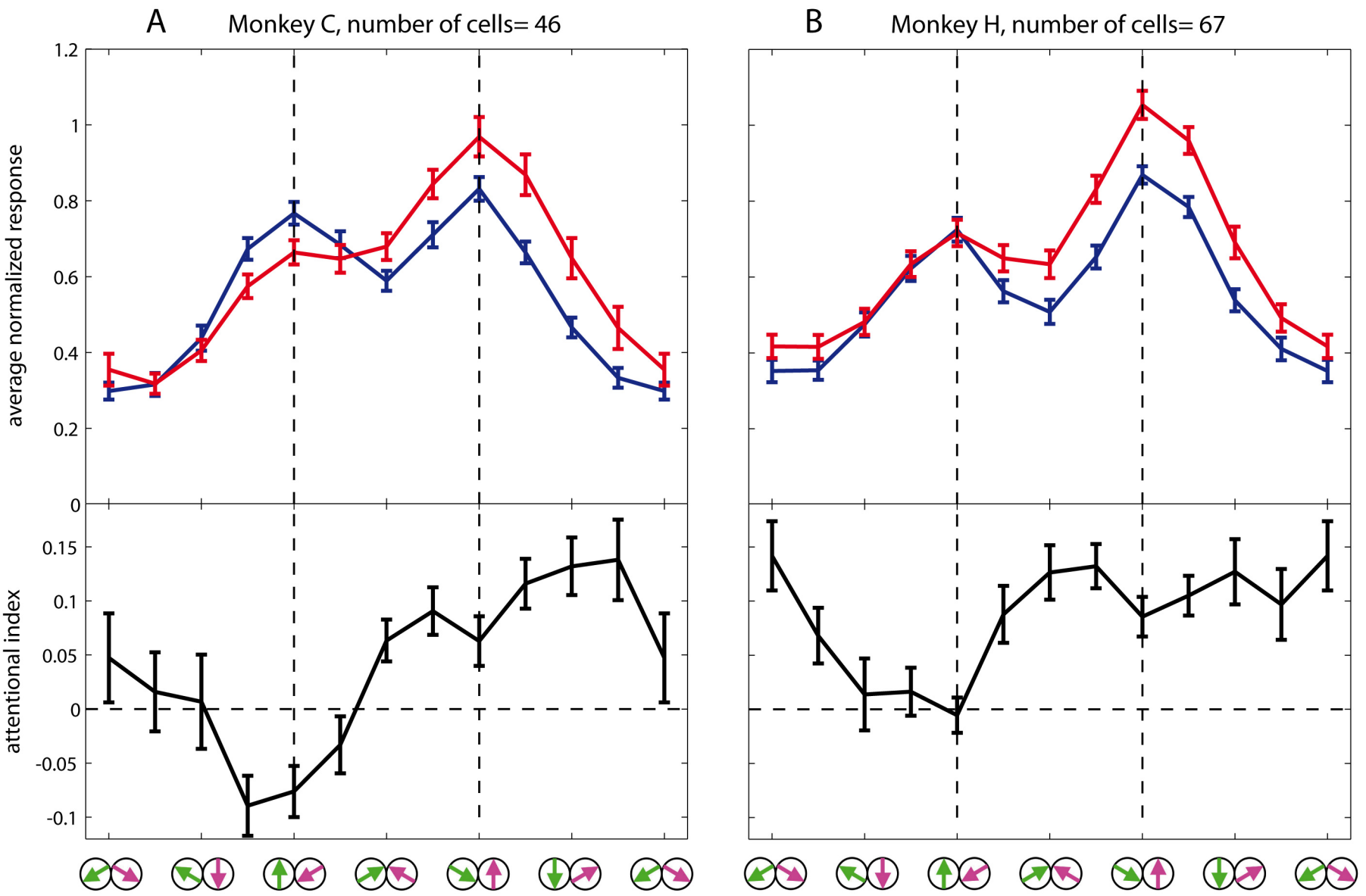

Supplement: S8 Fig — Layout is similar to the one used in Fig 3. The AI curve of monkey H compared with that of monkey C showed a stronger enhancement in most of the stimulus configurations. On the other hand, the suppression effect in monkey C was highly significant, whereas monkey H showed on average no suppression. Note though a large variability of the modulation effect in both monkeys when the direction of the attended pattern was close to antipreferred. As it was suggested by Khayat and colleagues (J Neurosci. 2010), the modulation in such configurations may be caused by FBA differentially modulating the strength of direction-selective inputs carrying signals from the 2 patterns into the recorded neurons. The AI was on average near zero in both monkeys when the antipreferred pattern is attended and the other pattern moves 120° apart (third point from the left), exactly the same as one observed by Khayat and colleagues for a similar configuration (see their Fig 7C). AI, attentional index; FBA, feature-based attention. (PDF) [file pbio.3000387.s012.pdf]

# Population activity profiles, number of cells= 88

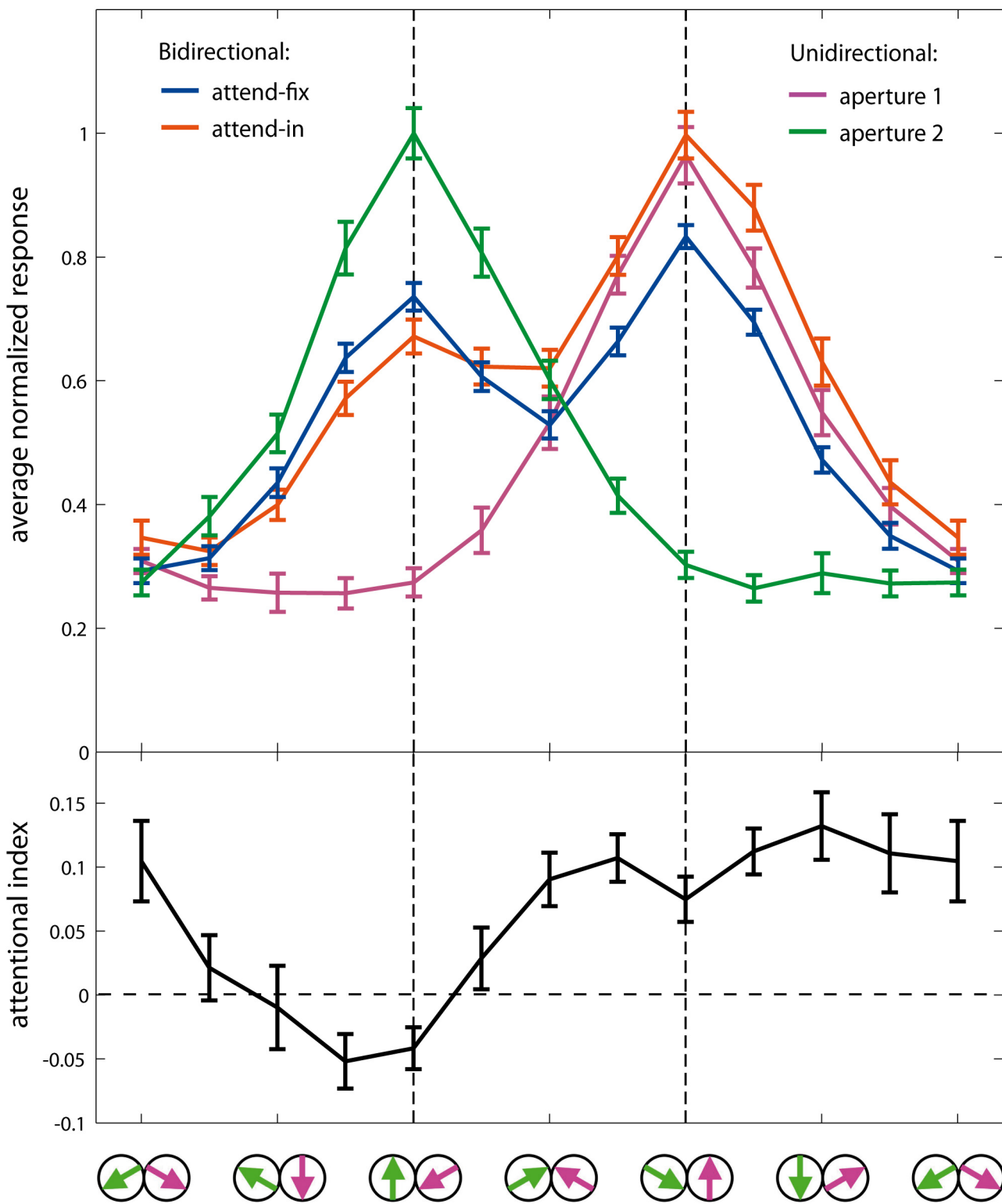

Supplement: S9 Fig — This subpopulation strongly overlaps with the 1 of 89 neurons depicted in Fig 3. The layout of the upper panel is similar to Fig 2A and 2B but combines all 4 average tuning curves in 1 plot. Fitting of each of unidirectional responses by single Gaussians shows that the tuning widths are very close to 45° (in terms of parameter b of Eq 3, median across cells). Comparison of the unidirectional and bidirectional (attend-fix) profiles show, in general, a quasilinear summation of the 2 component responses with some nonlinear interactions (like repulsion, width reduction, and unequal weighting of the peaks). The lower panel depicts the modulation profile, attend-in versus attend-fix (only bidirectional conditions) with error bars representing ±1 SEM taken across the 88 cells. As in the other subpopulations, the AI curve shows a trough at a point where the preferred direction is attended (compare with Fig 3, S8 Fig and S10A Fig). AI, attentional index. (PDF) [file pbio.3000387.s013.pdf]

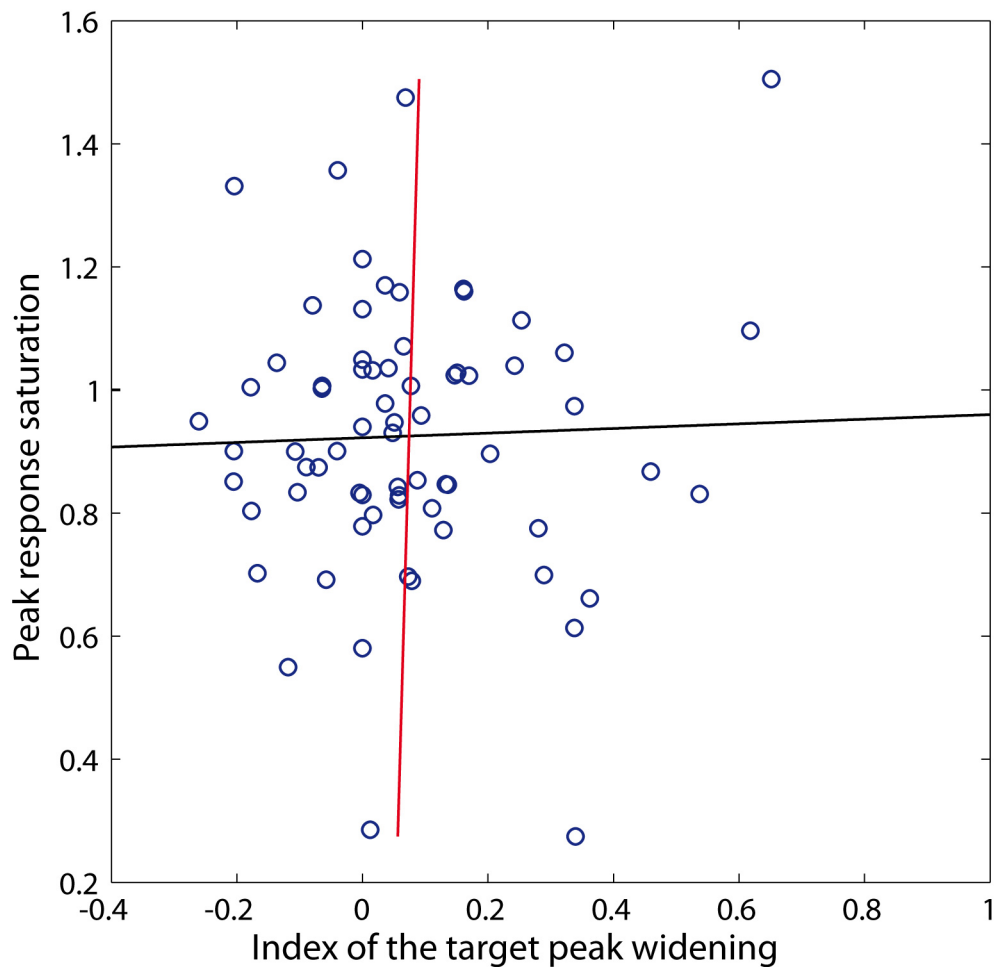

Supplement: S12 Fig — The peak response saturation was calculated for each cell as a ratio of maxima across 6 points: attend-in responses to preferred-60°… preferred+90° (aperture 1) in the numerator; 3+3 peak responses in the 2 attend-fix unidirectional conditions (preferred ±30° in aperture 1 or 2) in the denominator. The results show no correlation (r = 0.038; p = 0.756). (PDF) [file pbio.3000387.s016.pdf]

**A**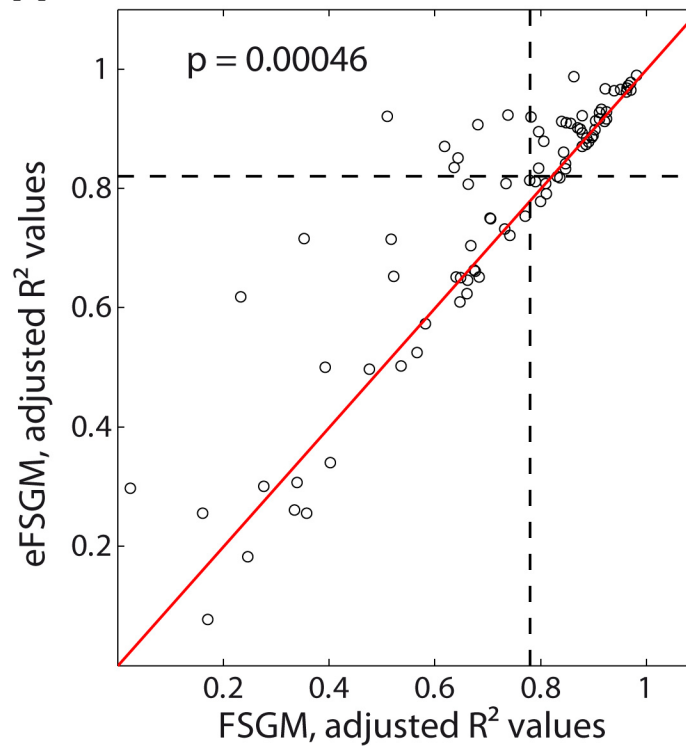**B**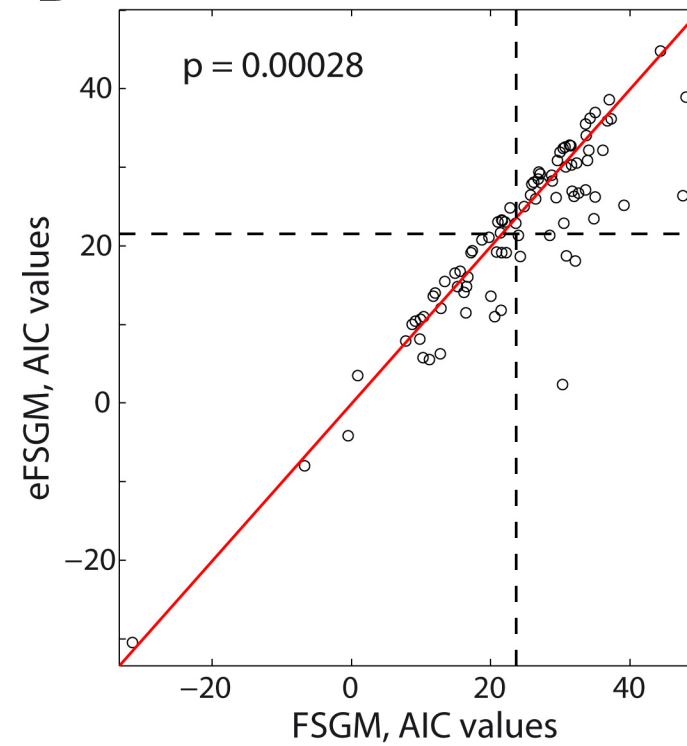**C**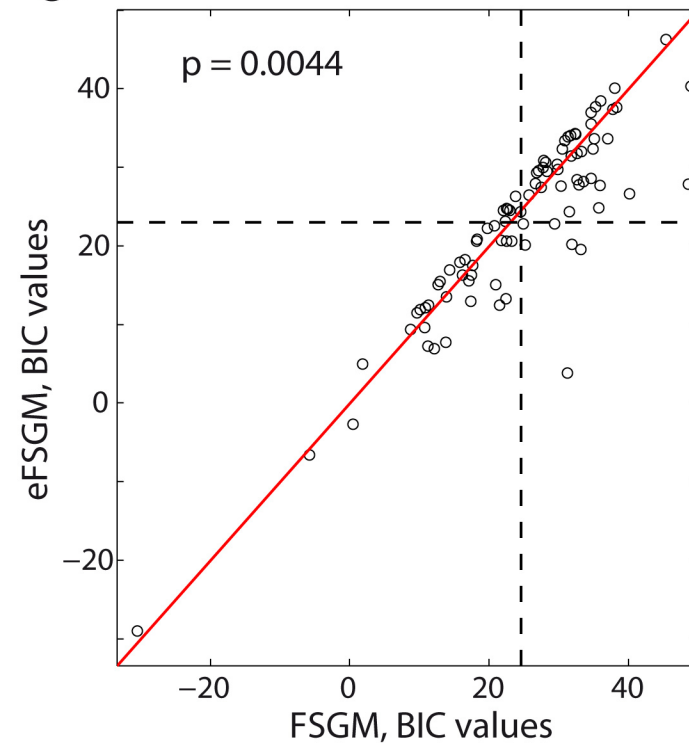

Supplement: S13 Fig — (A) adjusted R2, (B) AIC, and (C) BIC; for their definitions, see Materials and methods, and see S2 Data for the numerical values. The diagonal red line in each plot depicts points of equal goodness. Vertical and horizontal dashed lines depict the median (in A) or the mean (in B and C) of the criterion across cells. Performance of the 2 models was compared by Wilcoxon sign rank test across adjusted R2 values and by paired two-sided t test across AIC or BIC values; the calculated p-values are displayed on the respective plots. All 3 comparisons show a significant difference between the prediction qualities by the 2 models in favor of the eFSGM, accounting for de-aligned feature attention. AIC, Akaike information criterion; BIC, Bayesian information criterion; eFSGM, extended FSGM; FSGM, feature-similarity gain model. (PDF) [file pbio.3000387.s017.pdf]

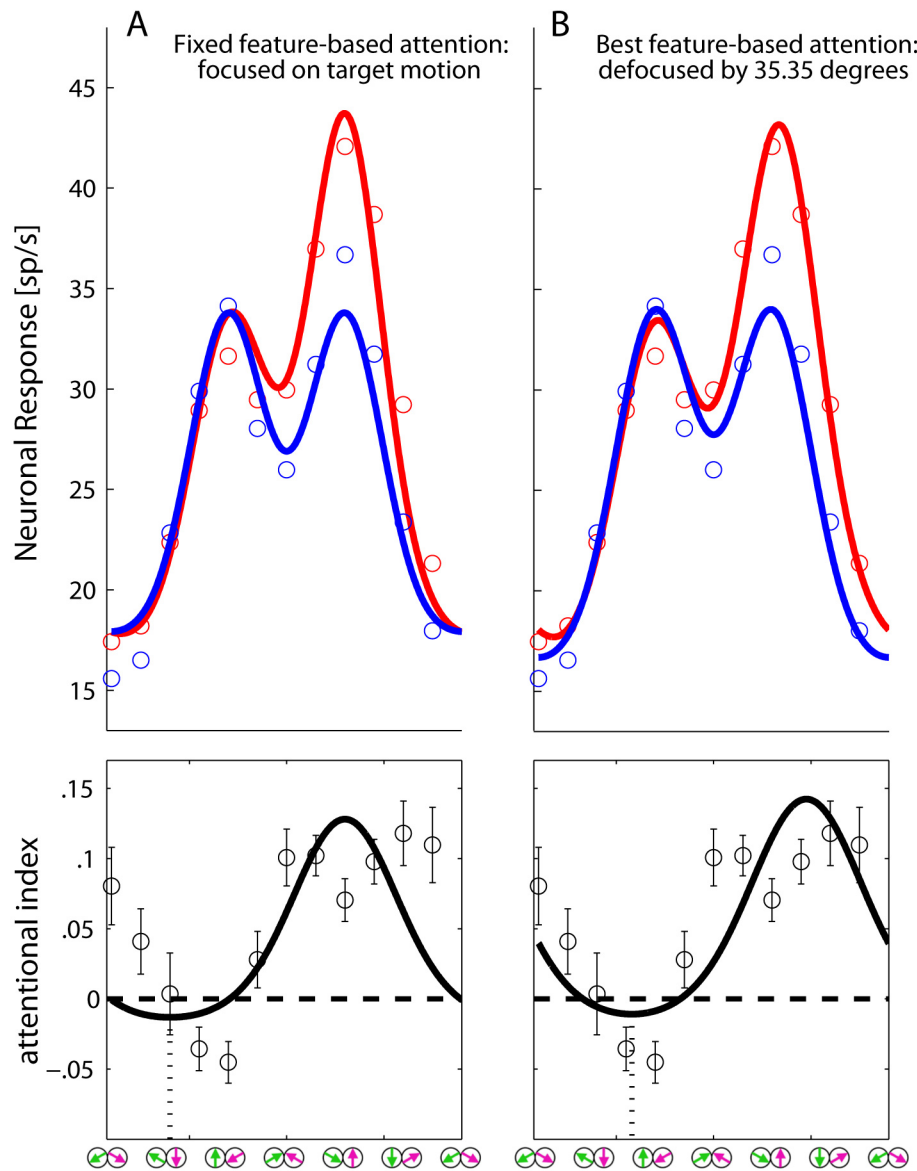

Supplement: S14 Fig — Both fits assume a modulated baseline. Further assumptions: (A) attention is focused on the target; (B) attention may focus off-target. In the latter case, therefore, there is an additional free parameter of the FBA defocus. Note an increased goodness (adjusted R2) of 0.958 in the model B compared with that of 0.931 in A. Because we used the mean firing rates (blue and red circles) across neurons rather than fitting each neuron individually, the goodness of fit is not directly comparable to that of the model fits presented in Fig 4. See S2 Text for further explanations. The NMoA, like the extended FSGM, provides significantly better fits if the defocus of FBA is allowed. FBA, feature-based attention; FSGM, feature-similarity gain model; NMoA, normalization model of attention. (PDF) [file pbio.3000387.s018.pdf]

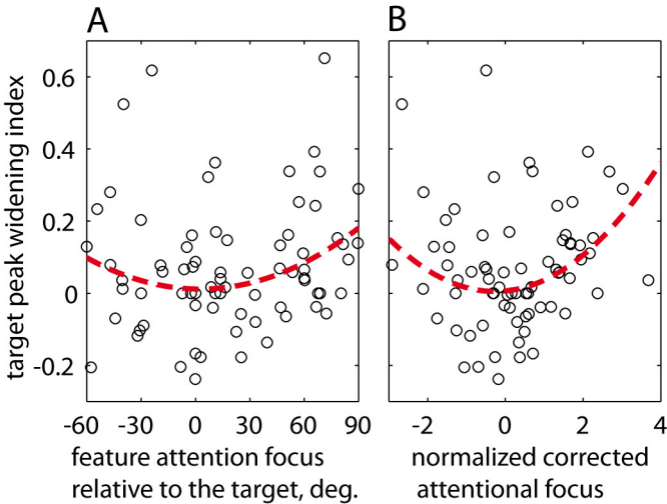

Supplement: S15 Fig — Seventy-four cells with reliably predicted values are included. (A) Second-order polynomial fit to this distribution is depicted by dashed curve. This fit includes a significant square coefficient (considering the 95% confidence interval) and accounts for about 25% of the data variance (r2 = 0.247). (B) Scatter plot representing the target peak widening indices as a function of the adjusted focus of FBA values. The latter consider variation of tuning width across neurons and the peaks repulsion effect. Here, the second-order polynomial fit accounts for 35% of the data variance. See S2 Text for further explanation. FBA, feature-based attention. (PDF) [file pbio.3000387.s019.pdf]

## Performance, attend-in condition

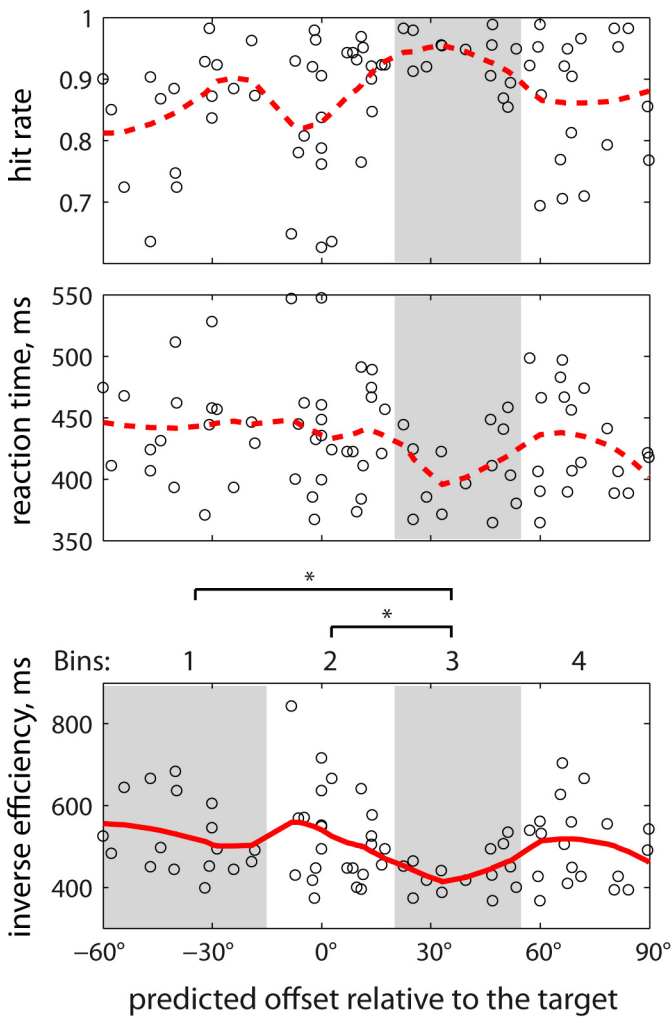

Supplement: S16 Fig — Upper panel depicts HR (ratio of correct trials excluding fixation errors); middle panel depicts RT, an interval (ms) between the target change and the lever release as functions of distance between the attentional focus and the target feature. Open circles denote the measured (fitted) points (for the values, see sheet Fig 6A in S1 Data); red dashed curves are locally weighted linear regressions to smooth the data. The 2 measures show the highest performance when the highest attention gain peaked at about 30° to 35° off-target, away from the distractor direction. The HR curve shows the second maximum at a negative angle (about 25° off-target in the direction of distractor). Lower panel plots IE (solid red curve) calculated as the reaction time normalized by the hitrate: IE = RT ÷ HR (see Romei and colleagues, Curr Biol. 2009). The predicted offset values were binned in 4 bins (approximately 35° wide, containing similar cell numbers per bin). The lowest IE (highest performance) took place at moderate positive de-alignments (third bin, 20°–55°), which was significantly different from the bins 1 and 2 (p < 0.05, Kruskal-Wallis test with multiple comparisons of mean ranks). When the attended feature was close to the target or de-aligned in the direction of the distractor, the performance was highly variable and on average lower than when the offset was away from the distractor. HR, hit rate; IE, inverse efficiency; RT, reaction time. (PDF) [file pbio.3000387.s020.pdf]

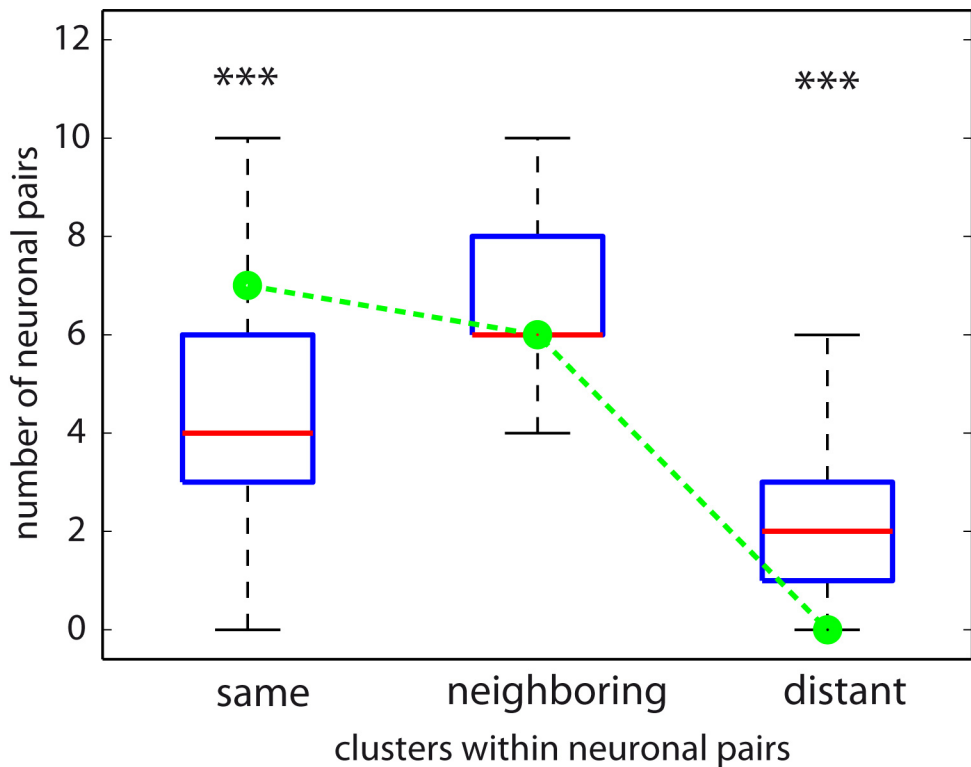

Supplement: S17 Fig — The green dashed curve shows the actual distribution (7, 6, and 0 pairs, respectively; for the values, see sheet Fig 6A in S1 Data). The box plot presents a distribution of shuffled pairs in which the cluster numbers of the first unit in each pair were randomly permuted 1,000 times. The red mark shows the median, edges of the box show the 25th and 75th percentiles, and the whiskers extend to the most extreme data points of the latter distribution (excluding outliers which are not shown). We tested if the shuffled data are distributed with the median equal to the actual numbers. The sign-test showed highly significant deviation from the respective medians in the “same” and “distant” groups (p < 0.0001), whereas it did not reach significance in the “neighboring” group. Therefore, the simultaneously recorded pairs of neurons are more similar in their attentional focus location than expected by chance. (PDF) [file pbio.3000387.s021.pdf]
