# Supplementary material for: Strategic deployment of feature-based attentional gain in primate visual cortex
Source: PLoS Biol. 2019 Aug 6;17(8):e3000387. doi: 10.1371/journal.pbio.3000387 (PMC6684042; doi:10.1371/journal.pbio.3000387)
Supplement: S10 Fig — (A) Normalized response profile (upper panel) and modulation profile, attend-in versus attend-fix (lower panel). The layout is similar to Fig 3. Neuronal responses for a given neuron were normalized to the highest firing rate in the attend-fix bidirectional condition in that neuron and aligned to the preferred direction. Error bars represent ±1 SEM taken across all cells. Modulation ratios for the conditions with the preferred stimulus in the RF are given in blue, the peak modulation ratios in red. The insertion shows mean ranks of modulation indexes (attend-in versus attend-fix) across 5 stimulus conditions close to the “preferred direction attended.” The conditions were compared by nonparametric Friedman's test with a follow-up multiple comparison test (see sheet S10 in S2 Data). Error bars denote 95% confidence intervals of the estimated mean ranks. The testing shows that 2 conditions (marked by red error bars with the respective p-values) when attention was somewhat away from the preferred direction have mean ranks significantly different from the condition in which just the preferred direction was attended (marked by blue error bars and dotted lines). The median goodness of the fits (adjusted R2 values) for the 113 accepted cells was 0.91. (B–D) Attentional modulation observed when the preferred direction was presented in the RF across the complete data set. The histograms show distribution of response changes caused by spatial (B), feature-based (C), and combined (spatial+feature, D) attention, respectively. Binning of the x-axis is according to the AIs. Bars include 2 differently colored stacks for the data from the 2 animals: brown for monkey C and gray for monkey H. On average spatial attention modulated responses by 16% (p < 0.0001, paired two-tailed t test), which is in agreement with earlier reports: Treue and Martinez Trujillo (Nature, 1999): 12%; Katzner, Busse and Treue (Front Syst Neurosci. 2009): 18%. The average modulation by FBA is 6% (p < 0.01), [file pbio.3000387.s014.pdf]

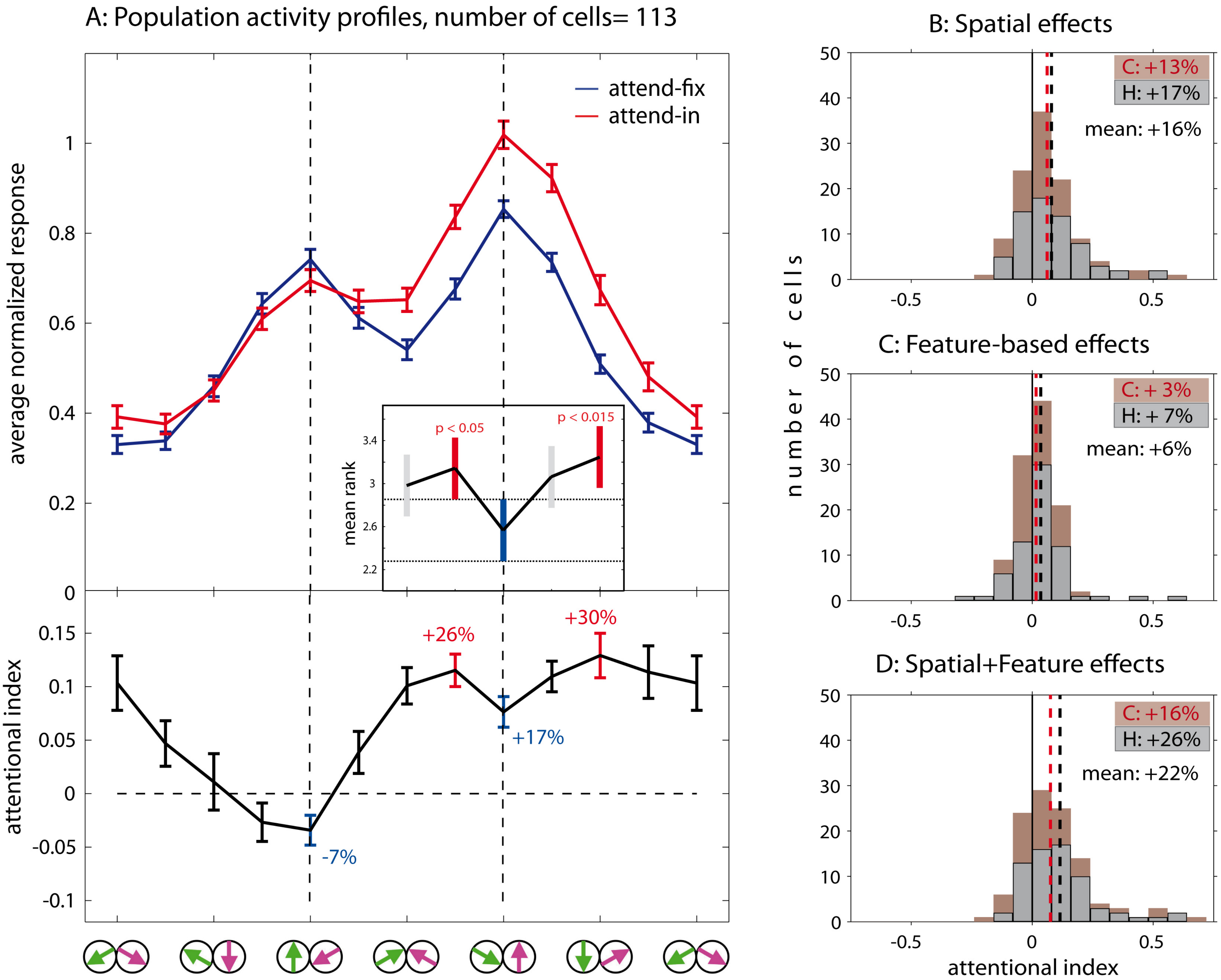
A: Population activity profiles, number of cells= 113
attend-fix
attend-in
average normalized response
1
0.8
0.6
0.4
0.2
0
mean rank
3.4
3
2.6
2.2
p < 0.05
p < 0.015
attentional index
0.15
0.1
0.05
0
-0.05
-0.1
+26%
+30%
+17%
-7%
B: Spatial effects
C: +13%
H: +17%
mean: +16%
C: Feature-based effects
C: + 3%
H: + 7%
mean: +6%
D: Spatial+Feature effects
C: +16%
H: +26%
mean: +22%
number of cells
50
40
30
20
10
0
-0.5
0
0.5
attentional index
