# Supplementary material for: Strategic deployment of feature-based attentional gain in primate visual cortex
Source: PLoS Biol. 2019 Aug 6;17(8):e3000387. doi: 10.1371/journal.pbio.3000387 (PMC6684042; doi:10.1371/journal.pbio.3000387)

Strategic deployment of feature-based attentional gain in primate visual cortex - Vladislav Kozyrev, Mohammad Reza Daliri, Philipp Schwedhelm & Stefan Treue, PLoS Biology, 2019

S11 Fig

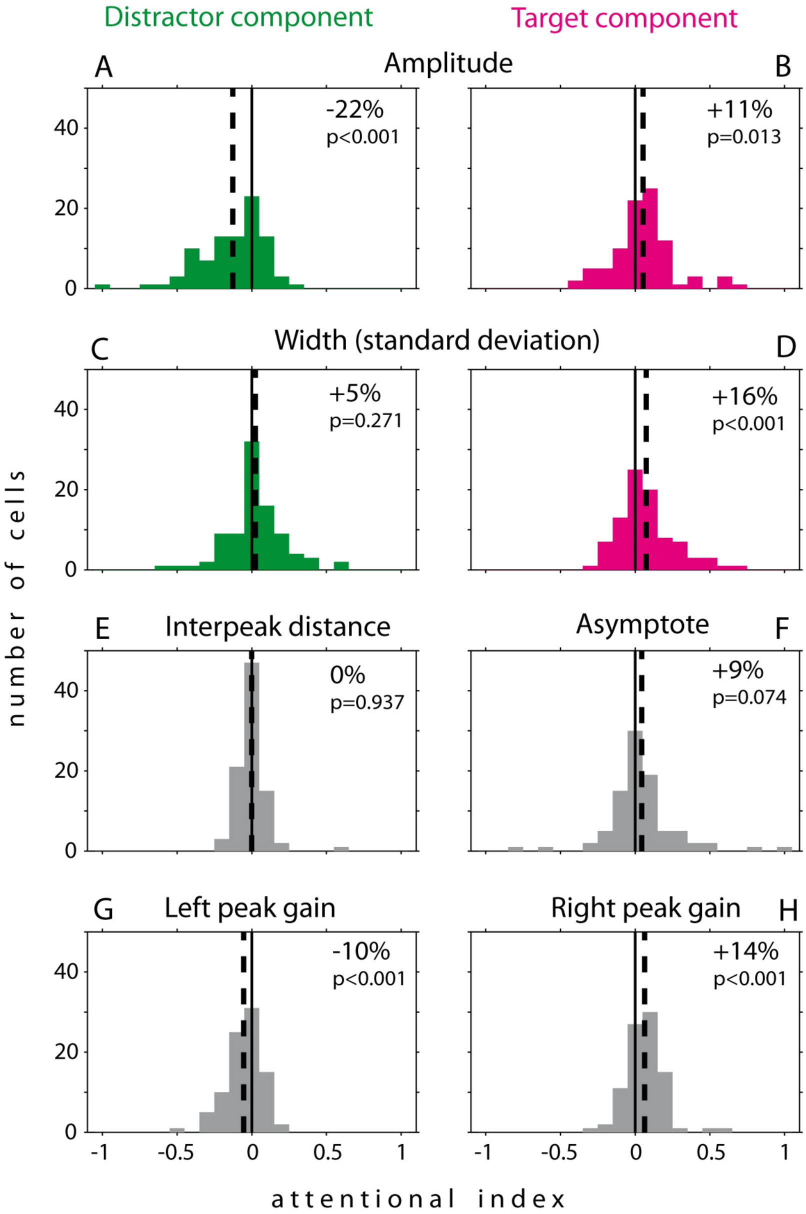

Supplement: S11 Fig — The individual histograms plot the attentional modulation of the amplitudes of the 2 Gauss functions (A, B), widths (standard deviations) of the 2 Gauss functions (C, D), distances between the centers of the 2 Gauss functions (E), asymptotic values of the fits in the 2 conditions (F). Histograms B and D denote the parameters of the component Gaussian corresponding to the target pattern, histograms A and C denote those of the distractor pattern. Mean indices are marked by vertical dashed lines and given in the insertions to the histograms together with the respective p-values (paired t test). (G, H) Relationship between the fitted peak firing rates in attend-in versus attend-fix conditions across the population of 89 neurons. Note that, unlike panels A and B, both of these histograms compare values relevant to both Gaussian components. In this case, the right peak gain (H) and the left peak gain (G) show the modulation ratios of, respectively, +14% and −10%, which are close to those estimated by our point-by-point responses averaging. (PDF) [file pbio.3000387.s015.pdf]
